# Encroachment diminishes herbaceous plant diversity in grassy ecosystems worldwide

**DOI:** 10.1111/gcb.16300

**Published:** 2022-07-11

**Authors:** Jakub D. Wieczorkowski, Caroline E. R. Lehmann

**Affiliations:** ^1^ School of GeoSciences The University of Edinburgh Edinburgh UK; ^2^ Tropical Diversity Royal Botanic Garden Edinburgh Edinburgh UK

**Keywords:** biodiversity, encroachment, grassland, grassy ecosystem, herbaceous, land‐use change, meta‐analysis, savanna, woody cover

## Abstract

Woody encroachment is ubiquitous in grassy ecosystems worldwide, but its global impacts on the diversity of herbaceous plants that characterise and define these ecosystems remain unquantified. The pervasiveness of encroachment is relatively easily observed via remote sensing, but its impacts on plant diversity and richness below the canopy can only be observed via field‐based studies. Via a meta‐analysis of 42 field studies across tropical to temperate grassy ecosystems, we quantified how encroachment altered herbaceous species richness, and the richness of forbs, C_3_ graminoids and C_4_ graminoids. Across studies, the natural logarithm of the response ratio (lnRR) of herbaceous species richness ranged from −3.33 to 0.34 with 87% of encroached ecosystems negatively impacted. Assessment of the extent of encroachment, duration of encroachment, mean annual rainfall, latitude, and continent demonstrated that only extent of encroachment had relevance in the data (univariate model including a random effect of study explained 45.4% of variance). The global weighted mean lnRR of species richness decreased from −0.245 at <33% of woody cover increase, to −0.562 at 33%–66%, and to −0.962 at >66%. Continued encroachment results in substantial loss of herbaceous diversity at medium and high extents, with a loss of richness that is not replaced. Although all functional groups are significantly negatively impacted by encroachment, forb richness is relatively more sensitive than graminoid richness, and C_4_ graminoid richness relatively more than C_3_ graminoid richness. Although no geographic or climatic correlates had relevance in the data, encroachment as an emergent product of global change coalesces to decrease ground layer light availability, lead to loss of fire and grazers, and alter hydrology and soils. Encroachment is accelerating and grassy ecosystems require urgent attention to determine critical woody cover thresholds that facilitate diverse and resilient grassy ecosystems.

## Graphical abstract

Woody encroachment is ubiquitous in grassy ecosystems worldwide but its global impacts on the diversity of herbaceous plants that characterise and define these ecosystems remain unquantified. Via a meta‐analysis across tropical to temperate grassy ecosystems, we show that encroachment leads to extensive declines in herbaceous species richness, and forb and graminoid functional groups, with declines more pronounced with increasing extent of encroachment. Encroachment can be viewed as a universally threatening process to grassy ecosystems and environmental policies that account for the unique ecology of these ecosystems will be vital to preserving grassy ecosystem biodiversity and integrity in the coming century.

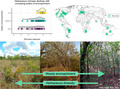

## 1 INTRODUCTION

Grassy biomes span approximately 40% of the global land surface and are geographically extensive across temperate and tropical regions (Scholes & Archer, 1997), defined by a continuous grassy ground cover and discontinuous tree cover (Lehmann et al., 2019; Ratnam et al., 2011; Sankaran et al., 2005). Grassy biomes are critical to livelihoods, economy, culture, biodiversity, and Earth System functioning and deliver a wide range of supporting, provisioning, regulating, and cultural services (Murphy et al., 2016; Osborne et al., 2018; Van Vooren et al., 2018). Studies the world over and at varied spatial and temporal scales document woody cover increases in grassy ecosystems (Knapp et al., 2008; Rosan et al., 2019; Stevens et al., 2017), a phenomenon described as encroachment (Archer et al., 2017). Rapid and accelerating encroachment is globally ubiquitous although remains an under‐appreciated impact of global change (Eldridge et al., 2011; Stevens et al., 2017). Encroachment likely has a profound impact on biodiversity in grassy ecosystems but curiously understanding of impacts remains local and regional. With encroachment, loss of herbaceous diversity from grassy ecosystems can be severe (e.g., Abreu et al., 2017) and even with active restoration grassy ecosystems may not recover for centuries (Nerlekar & Veldman, 2020). Moreover, changes to vegetation structure of grassy ecosystems will have implications for the invertebrate, mammal, reptile and bird habitats they provide (Parr et al., 2012; Sirami & Monadjem, 2012).

Most plant diversity in grassy ecosystems is in herbaceous plants (vascular plants with non‐woody stems) (Perez‐Garcia & Meave, 2006), with forb diversity (flowering plants of self‐supporting growth form, excluding graminoids) especially high, followed by graminoids—grasses, rushes, and sedges (Bond & Parr, 2010). Grassy ecosystems are often juxtaposed to biodiverse forests and, thus mistakenly considered degraded (Griffith et al., 2017; Ratnam et al., 2011). Numerous grassy ecosystems are biodiversity hotspots such as the Cerrado of Brazil and long‐leaf pine savanna of southeastern USA, both threatened by encroachment (Lehmann & Parr, 2016; Rosan et al., 2019). Further, Murphy et al. (2016) found that there can be a higher diversity of mammalian megafauna in tropical grassy ecosystems than in forests of similar climate. Similarly, invertebrates may be of higher diversity in tropical grasslands than forests (Bond & Parr, 2010). Grassy biomes were formed by complex evolutionary and ecological interactions among plants and disturbance resulting in unique assemblages (Veldman et al., 2015) representing complex food webs and habitats (Anderson et al., 2016). The concept of grassy ecosystem as a successional stage preceding a forest climax is misleading, and the two are better viewed as alternate states maintained by disturbance‐centred feedbacks (Bond & Parr, 2010), where across much of their range, forest and grassy biomes are bistable (Staver et al., 2011). The contrasting processes of disturbance and shade lead to divergent assemblages in grassy and forest ecosystems with few species common even in locally adjacent areas (Bond, 2016; Hoffmann et al., 2009), suggesting the integrity of grassy ecosystem plant diversity and ecosystem function is dependent on canopy openness (Bond, 2016; Lehmann & Parr, 2016).

Although some aspects of climate change could inhibit encroachment, such as regional rainfall reductions, higher temperatures or increased El Niño intensity leading to changed patterns of drought, fire, and tree mortality (Lehmann & Parr, 2016), widespread increases in woody cover in grassy ecosystems are predicted (Higgins & Scheiter, 2012). Encroachment has been widespread in grassy ecosystems globally for more than 100 years, although with regional variations in magnitude (Archer et al., 2017; Stevens et al., 2017). Numerous factors combine to produce the observed trajectories of encroachment. Increasing atmospheric CO_2_ concentration (p[CO_2_]) alters the competitive balance of woody plants and grasses (Bond & Midgley, 2012); improves woody plants' ability to recover from fire and herbivory (Kgope et al., 2010) and nitrogen and water‐use efficiencies (Leakey et al., 2009); and extends growing season (Reyes‐Fox et al., 2014). Other suggested factors include changing rainfall and temperature patterns (Fensham et al., 2005), or deposition of atmospheric nitrogen (Köchy & Wilson, 2001; Wigley et al., 2010). Regionally, increases in heavy grazing reduce the competitive effect of grasses as livestock preferentially feed on herbaceous plants while woody plants are defended via chemical (e.g. production of secondary metabolites) and physical (e.g. spinescence) traits; and with lower herbaceous biomass, woody plant growth is supported by increased resource availability (Coetzee et al., 2008; Estell et al., 2012; O'Connor et al., 2014; Walker et al., 1981). However, grazing abandonment can also result in encroachment (Guardiola et al., 2013; Kinnebrew et al., 2020), whereas reductions in browsers lead to encroachment through reduced pressure on adult plants and seedling regeneration (O'Connor et al., 2014). Land fragmentation disrupts fire regimes and restricts animal movements (Rosan et al., 2019; Stevens et al., 2017) with the global burned area having declined by 25% in the last two decades, and where the largest decreases occurred in tropical grassy ecosystems (Andela et al., 2017). Long‐term fire exclusion experiments demonstrate encroachment (Woinarski et al., 2004; Zhou et al., 2022), while fire suppression policies have favored encroachment in regions such as the Cerrado (Abreu et al., 2017). By altering light and water availability, fire regimes, herbivory, soil characteristics, or competition for space, encroachment can affect different plant taxonomic and functional groups.

A number of field studies described negative impacts of encroachment on herbaceous richness and diversity due to shading and litter accumulation, and competition from forest‐adapted species and invasive weeds (Abreu et al., 2017; Parr et al., 2012), alongside turnover in functional groups (Pilon et al., 2021). Many forb species in grassy ecosystems are suited to open‐canopy conditions and frequent disturbance, whether in the form of herbivory or fire (Siebert & Dreber, 2019; Uys, 2006). C_4_ grasses are physiologically adapted to open and warm conditions and most are unlikely to persist in shaded conditions (Pilon et al., 2021). Grassy ecosystem specialists from temperate to tropical environments have undergone local extinction with encroachment with a minimal turnover to a shade‐tolerant composition (Abreu et al., 2017; Parr et al., 2012; Peterson & Reich, 2008). Importantly, biodiversity loss also includes threatened species (Costello et al., 2000). Where encroachment correlates with a loss of fire or herbivory, disturbance‐adapted species such as those that resprout from belowground storage organs are also likely to be lost (Bond, 2016). Nevertheless, the extents of encroachment that equate to declines in herbaceous richness are yet to be clearly assessed.

Extensive encroachment likely results in losses and homogenisation of unique herbaceous diversity. A previous global meta‐analysis examining encroachment impacts on plant diversity found no clear response of vascular plant richness to encroachment although it did not specifically examine grassy ecosystems (by examining both shrub‐ and grass‐dominated ecosystems of mean annual rainfall [MAR] ≤ 850 mm), nor herbaceous plants (Eldridge et al., 2011). A meta‐analysis of North American grassy ecosystems (of MAR ≤ 1100 mm) found plant species richness declined in response to encroachment but suggested the strong negative response may be unique to North America (Ratajczak et al., 2012). Surprisingly, there remains no synthetic understanding of encroachment impacts on specifically herbaceous richness and functional diversity in grassy ecosystems, and how that varies with extent of encroachment, hampering efforts to both predict and mitigate ecosystem‐level changes caused by encroachment. Similarly, there is no quantification of encroachment impacts on herbaceous plants representing grassy ecosystems worldwide from tropical to temperate regions, and high‐rainfall areas. To mitigate the degradation of grassy ecosystems via encroachment, we need to understand how extent and duration of encroachment exert control on the richness of the herbaceous plants that define grassy ecosystems, and how this differs with climate and geography. Here, we aim to quantify how encroachment (defined as increases in woody cover) relates to herbaceous species richness and the richness of functional groups of forbs, C_3_ graminoids and C_4_ graminoids in grassy ecosystems via a meta‐analysis of 42 studies spanning six continents.

## 2 METHODS

### 2.1 Literature search and screening

We conducted a meta‐analysis of encroachment impacts on herbaceous richness in tropical to temperate grass‐dominated ecosystems defined as having a continuous grassy ground layer and a variable woody component (Ratnam et al., 2011). A threshold of mean annual temperature ≥ 0°C was used for studies to be included. Hyper‐arid ecosystems where the grassy ground layer is sparse were not incorporated as these did not meet the criteria of a grass‐dominated ground layer.

Our approach required a comparison between encroached and unencroached sites with an ecosystem‐level difference between them (i.e. studies were excluded if they compared richness below versus away from a woody canopy). Studies either (1) reported a difference in woody cover between two proximal ecosystems where encroachment had taken place in one or (2) a single ecosystem was measured over time before and after encroachment. For a study to be included, herbaceous species richness had to be documented where this could include graminoids, forbs, ferns, herbaceous climbers, and sub‐shrubs (defined as <1 m height at maturity and largely herbaceous such as geoxyles). If data were provided or able to be provided, the richness of forbs and graminoids (with division to C_3_ and C_4_) was additionally calculated.

To account for nomenclature variation describing grassy ecosystems, studies were found through Web of Science (WOS) with topic search (TS) criteria: **TS** = (“grassy ecosystem$” OR grassland$ OR savanna$ OR woodland$ OR prairie$ OR cerrado$ OR steppe$ OR veld$ OR meadow$) *AND* **TS** = (encroach* OR invasion$ OR invad* OR thicken* OR thicket* OR (fire$ AND [tree$ OR shrub$ OR woody])) *AND* **TS** = (herb$ OR herbaceous OR understor* OR grass OR grasses OR graminoid$ OR forb$ OR groundlayer* OR “ground layer” OR plant$) *AND* **TS** = (diversity OR richness). The search was done for studies published up until 30 June 2021.

Our WOS search returned 3270 studies. Studies were excluded where other treatments or land‐use effects would compromise direct estimation of encroachment impacts (List S1). Multiple data points from a single publication were incorporated if comparisons were made among distinct levels of woody cover, different durations of encroachment were reported, or observations were made in substantively different sites. In sum, 37 studies fulfilled all criteria. An additional five studies were added based on our knowledge of the literature where studies were not returned in our WOS search due to publishing date, missing keyword, or format (e.g., a PhD thesis). The list of studies used is provided in List S2.

### 2.2 Variable selection and hypotheses

From each study, we compiled the lnRR (natural logarithm of the response ratio) of herbaceous species richness related to encroachment, the mean of which we hypothesised to be negative. Encroachment impacts have been suggested to increase with increasing extent of encroachment (Abreu et al., 2017), and hence extent of encroachment was calculated as a categorical variable where we expected lnRR to decrease with increasing extent of encroachment. Other factors likely moderate any relationship between encroachment and herbaceous species richness including duration of encroachment, MAR, latitude, and continent (Table 1). Although grazing regime and soil type would be further important variables which could be analysed, data were not comparable across studies. We provide additional notes on grazing and soil along with the full dataset compiled for this meta‐analysis which is available on figshare (Wieczorkowski & Lehmann, 2022). For each data point, plot size, sampling effort, and cause of encroachment (as determined by authors) were compiled although not incorporated in formal analyses and were used to assess potential sources of bias.

**TABLE 1:** Summary of variables extracted from each study and used in the analyses and accompanied by the main hypotheses tested.

Variable type	Variable	Abbrev.	Description	Main hypotheses
*Response variable*	lnRR of species richness	lnRR	The natural logarithm of the response ratio of species richness between the areas of higher and lower woody cover	lnRR is lower than 0; grassy ecosystem biodiversity requires canopy openness and thus richness should be on average negatively impacted
*Fixed effects*	Extent of encroachment	extent	Relative percent cover change in the extent of encroachment; classified as low (<33% change), medium (33%–66% change), high (>66% change)	lnRR decreases with increasing extent of encroachment; we anticipate increasing severity of impact on richness with increasing extent of encroachment
	Duration of encroachment	duration	Short (≤30 years) or long (>30 years) category	lnRR decreases with increasing duration of encroachment; extinction of grassy ecosystem specialists is expected to become more common with time, diminishing total herbaceous richness
	Mean Annual Rainfall	MAR	Mean Annual Rainfall [mm]	lnRR decreases with increasing Mean Annual Rainfall; at low rainfall, some environmental changes may be of benefit to species richness (Eldridge et al., 2011), while at higher rainfalls, the effect of encroachment is expected to be generally negative
	Latitude	latitude	Latitude of the study site; recorded in absolute numbers (no N/S differentiation)	lnRR decreases with decreasing latitude; tropical areas might be impacted more due to increased growth of woody plants
	Continent	continent	Location of study	lnRR varies across continents; continents differ in plant diversity and the nature of encroachment which can result in a variation of responses to encroachment
*Random effect*	Study	study	The study from which data come from	—

The response variable was **lnRR of herbaceous species richness** which is commonly applied in ecology for meta‐analyses due to the differences in methodology and characteristics of richness values among studies (Koricheva & Gurevitch, 2014) and here was calculated as ln(*richness at higher woody cover/richness at lower woody cover*). Positive values indicated an increase in richness with encroachment, and negative values a decrease in richness with encroachment. Previous meta‐analyses of plant species richness have used this effect‐size metric because it allows standardisation among studies and, its calculation does not require variance data (Hedges et al., 1999; Nerlekar & Veldman, 2020; Ratajczak et al., 2012). Where possible, we distinguished forbs and graminoids, and C_3_ and C_4_ graminoid species richness as division of these functional groups provides insights into whether responses to encroachment are uniform. lnRR was preferably calculated as mean species richness per unit area although it was calculated as a median/total species richness where a mean was unavailable. Species richness values were extracted from published text, figures or calculated from the datasets attached to studies, including classification of functional groups via online species catalogues (Encyclopedia of Life, 2021; JSTOR Global Plants, 2021; Plants of the World Online, 2021). Where values could not be distinguished, we requested data from authors with a 6‐week waiting period for reply.

**Extent of encroachment** was calculated as a relative difference in tree/shrub/woody cover over a given time period or location. To provide differentiation among the stages of potential encroachment impacts and where different studies inherently measured ecosystems at different points in a trajectory of ecosystem change, we divided the extent of encroachment into three categories of equal range based on the relative percent woody cover change. (1) Low, <33%—a small relative change in woody cover and light environment; (2) Medium, 33%–66%—a substantial increase in woody cover where usually some open canopy remains; and (3) High, >66%—woody cover significantly alters the ecosystem transforming light environments. For a subset of studies, woody cover as a % of the total area was not available and encroachment category was assigned based on available woody plant density or abundance, leaf area index data, descriptions, or via consulting the authors of the study directly. Woody cover was not equally described amongst studies, it could not be assessed on a continuous scale, and hence categorisation was important to not treat encroachment as equal across studies where the extent could range from just a few woody individuals appearing, to an area becoming 100% woody cover. The relative cover changes and their associated categories are illustrated by these examples—an increase from 0% to 70% falls into “high” while 30% to 40% into “low.”

Given the variability among studies, **duration of encroachment** was estimated as either the time period since the compared ecosystems last had a similar woody cover, or as the time period between sampling the same site before and after encroachment. Duration was divided into two broad categories of ≤30 years (short) and >30 years (long) as exact numbers were not always clearly reported but where the richness of grassy ecosystem specialist herbaceous species would be expected to decline slowly, while the recruitment of shade‐tolerant herbaceous species potentially benefiting from encroachment could appear much quicker and thus boosting richness at the beginning (Haugo & Halpern, 2007). Hence, the lnRR of species richness from short versus long periods of encroachment could have different responses.

In grassy ecosystems, **MAR** is a central determinant of primary productivity. At low rainfall, the impacts of encroachment can be diverse (Eldridge et al., 2011), whereas at higher rainfall impacts have been reported as increasingly negative (Ratajczak et al., 2012). However, grassy ecosystems can be found in regions with up to 3000 mm MAR, and it is unclear how MAR and encroachment impacts are related. MAR was extracted for each location from the studies and via WorldClim v.2.1 climate data for 1970–2000 (Fick & Hijmans, 2017).

**Latitude** is correlated with light availability, temperature (mean annual temperature and latitude were significantly correlated in our dataset and so latitude could be used as a proxy for temperature; Pearson's *r* = −0.78, *p*‐value < .001; Figure S1), temperature seasonality and primary productivity (Gillman et al., 2015). As woody species can grow faster in the tropical rather than temperate zones (Locosselli et al., 2020), and plant diversity increases toward the equator (Barthlott et al., 2007), we posited that similarly lnRR might be positively correlated with absolute latitude with negative encroachment impacts larger at lower latitudes.

It was anticipated **continent** could influence the response of herbaceous species richness to encroachment due to (1) the prevalence of C_4_ versus C_3_‐photosynthesising plants in, for example, Africa and Australia as opposed to Europe and Asia (Still et al., 2003) where C_4_ plants are likely more at risk from encroachment due to the adaptation to frequent fire disturbance and high light availability; (2) differences in the functional traits of woody encroachers and the rates of encroachment among continents (Stevens et al., 2017); and (3) differences in land‐use histories and drivers of encroachment. For example, encroachment due to grazing abandonment is common in Europe while fire suppression is considered an important cause of encroachment in the South American Cerrado (Abreu et al., 2017; Zehnder et al., 2020). Furthermore, Ratajczak et al. (2012) suggested that plant species richness responses to encroachment could be pronounced in North America due to its unique evolutionary history.

**Study** was included as a random effect in statistical models to account for the non‐independence of data in cases where multiple data points (of different duration categories/extents of encroachment/locations) were used from a single study.

### 2.3 Statistical analyses

Analyses were conducted using R v.4.0.2 (R Core Team, 2020). The studies included will reflect biases in the literature, and these studies inherently examined different numbers of sites, sampled in different ways with plots of different sizes. Before formal analyses, we first inspected normal distribution of response variables by plotting histograms and by checking the distribution of residuals with normal quantile‐quantile plots. We also checked whether including the five additional studies not returned by the Web of Science search impacted the distribution of lnRR of herbaceous species richness data. Furthermore, we conducted linear regressions of lnRR of herbaceous species richness and (1) method of lnRR calculation (mean, median, total)—because using lnRR with small sample sizes (such as totals) is more likely to produce bias (Lajeunesse, 2015); (2) plot size—to check the impact of the sampling area; and (3) cause of encroachment—to see if the main cause of encroachment in each study has an impact on the lnRR. Significance level for all analyses and tests in this paper was set at .05. Full description of results of these assumption checks, and all additional checks described in sections below (multicollinearity, homoscedasticity), sensitivity tests (for lnRR > −2; global average with equal weighting), and test for publication bias (fail‐safe N) are fully described in Appendix S1.

#### 2.3.1 Examining correlates of herbaceous richness response to encroachment

Linear mixed‐effects models from package *lme4* v.1.1‐23 (Bates et al., 2015) were applied to assess the impact of the above fixed effects (Table 1) on the lnRR of herbaceous species richness. Asia was represented by one data point and therefore removed from this analysis. Multicollinearity of five fixed effects in the base model was assessed with a variance inflation factor to identify correlations between variables assumed to be independent (Fox & Weisberg, 2019), which we further checked by assessing correlations among the individual pairs of each of the fixed effects (Table S1 and Figure S2). Model comparison was conducted with Akaike information criterion (AIC) using backward elimination selection which allows assessing the joint predictive ability of all variables and then removing the least significant variables early on (Chowdhury & Turin, 2020; Goodenough et al., 2012). Consequently, the model with the lowest AIC was chosen, with significance assessed by calculating model predictions with 95% confidence intervals (95% CIs) and checking the *p*‐value resulting from a likelihood ratio test (comparing the given model with a model with no fixed effects). The assumption of homoscedasticity was inspected by plotting residuals versus fitted values. Additionally, we ran separate models for each fixed effect (including a random effect of study) to check whether any have an impact when treated separately. To ascertain the robustness of results considering the slight left‐skewness of distribution of the lnRR of herbaceous species richness, we also conducted a sensitivity analysis removing values of lnRR < −2.

#### 2.3.2 Global weighted mean response of herbaceous richness to encroachment

We calculated a weighted global mean lnRR for each extent of encroachment that considers the variance associated with each study by applying a weighting to each value. In studies that report on one category of extent of encroachment but multiple locations or time points, we calculated averages. Using OpenMEE (Wallace et al., 2017), we performed a random‐effects meta‐analysis using Restricted Maximum Likelihood with inverse variance weighting with three categories reflecting the three levels of the extent of encroachment. As opposed to a fixed‐effects model, the random‐effects model within OpenMEE allows accounting for the real differences among studies as well as the random sampling variation among the outcomes of studies (Gurevitch & Nakagawa, 2015). Variance of lnRR was calculated for each study based on the reported sample sizes as *V*_lnRR_ = (*N*_hwc_ + *N*_lwc_)/(*N*_hwc_ × *N*_lwc_) where *N*_hwc_ is the number of plots at higher woody cover and *N*_lwc_—at lower woody cover. Inverted variance was used to calculate the weighting of individual data points, where studies with high sample sizes were given a higher weighting than studies with low sample sizes. Therefore, the averaging of multiple locations within one study (as mentioned above) was compensated through increased weighting. Given the high heterogeneity in lnRR detected between studies (i.e. high I^2^ values) for medium and high extents of encroachment in the meta‐analysis, we also checked results by applying equal weighting to each data point. To assess publication bias, we conducted the fail‐safe N analysis using Rosenberg (2005) method.

#### 2.3.3 Responses of forbs, C_3_ and C_4_ graminoids

As the components of herbaceous richness, we expected the functional groups of forbs, graminoids, C_3_ graminoids, and C_4_ graminoids to be negatively impacted by encroachment, but where forbs are more impacted than graminoids, and C_4_ graminoids more than C_3_ graminoids. Forbs can be more severely impacted due to their persistence life‐strategy involving belowground storage organs and where graminoids disperse and colonise new environments readily (Linder et al., 2018; Parr et al., 2012; Zaloumis & Bond, 2011). Meanwhile, C_4_ graminoids are more sensitive to light environments than C_3_ graminoids due to the morpho‐physiological constraints of C_4_ (Pilon et al., 2021). Therefore, one‐tailed one‐sample *t*‐tests were conducted, and we hypothesised the lnRR of each group to be lower than 0. Then, we quantified the differences between lnRR of forbs and graminoids, and lnRR of C_4_ and C_3_ graminoids using one‐tailed paired samples *t*‐tests.

## 3 RESULTS

**FIGURE 1:**
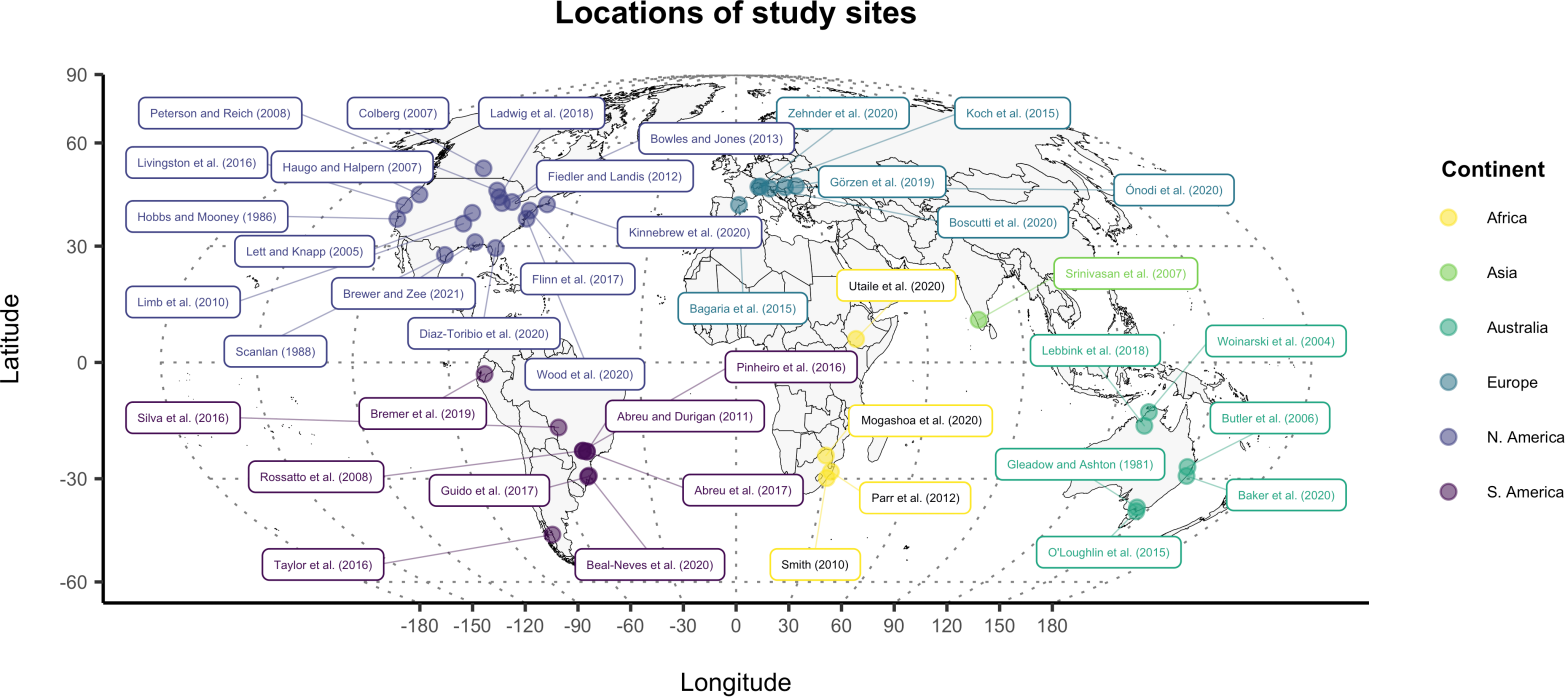
Map of study locations used in the meta‐analysis. Due to the high proximity of distinct locations within studies, the map shows only one geographic location per study. Full references are listed in List S2.

From 42 studies, we compiled 71 data points that calculated change in herbaceous species richness relative to encroachment (Figure 1, Table 2). At a functional group level, we further distinguished 47 data points estimating change in forb richness, 50 in graminoid richness, 29 in C_3_ graminoid, and 26 in C_4_ graminoid. The 71 lnRR values on herbaceous richness ranged from −3.33 to 0.34 and averaged −0.69 ± 0.09. In 87% of cases, the lnRR values were negative and indicated a reduction in herbaceous species richness with encroachment. Study locations were found in both hemispheres, as close to the equator as 2.94°S, to as far as 52°N with tropical, subtropical and temperate regions well represented. The largest number of points were found in North America (30), followed by South America (13), Europe (12), Australia (9), Africa (6) and Asia (1) (Figure 1). Most of the highly negative values were found in the Americas, however, this may be due to larger sample sizes and thus a higher probability of cases of extreme reduction in richness. MAR values ranged from 369 to 2700 mm with a median of 1041 mm, and with the lowest values (lnRR < −1) found in the range of 716–1400 mm. The main single causes of encroachment listed by authors of 42 studies were fire reduction (18), grazing decline (6), invasion (5; i.e. invasive species encroaching due to proximity of sources or unidentified causes), temperature rise (1), p[CO_2_] rise (1) or unclear (11).

**TABLE 2:** Statistical summary of lnRR of (a) herbaceous, (b) forb, (c) graminoid, (d) C_3_ graminoid, and (e) C_4_ graminoid species richness. Mean lnRR of each group was lower than 0; *p*‐values of one‐tailed one‐sample *t*‐tests: (a) <.001, (b) <.001, (c) <.001, (d) .009, (e) <.001

lnRR of species richness	(a) Herbaceous	(b) Forb	(c) Graminoid	(d) C_3_ Graminoid	(e) C_4_ Graminoid
Min–Max	−3.33–0.34	−3.64–0.23	−3.45–0.34	−2.08–1.68	−2.86–0.63
% negative	87%	89%	84%	69%	88%
Median	−0.48	−0.54	−0.46	−0.18	−0.55
Mean	−0.69	−0.77	−0.63	−0.32	−0.65
*n*	71	47	50	29	26

Based on the initial assumption checks, modelled response variables could be assumed to be normally distributed apart from the lnRR of herbaceous species richness which was slightly negatively skewed (Figure S3, Figure S4). The addition of five studies not returned by WOS did not impact the range, median or the mean of lnRR of herbaceous species richness (Table S2). There was no significant relationship between lnRR and (1) the method of richness calculation (*p*‐value = .082), (2) plot size (*p*‐value = .650), or (3) cause of encroachment (*p*‐value = .161) (Figure S5).

### 3.1 Effects of extent and duration of encroachment, climate, and geography on herbaceous richness

**FIGURE 2:**
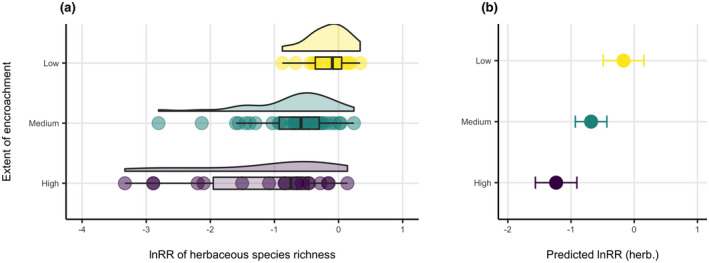
(a) Distribution of lnRR of herbaceous species richness at three extents of encroachment and (b) corresponding model predictions for the linear mixed‐effects model lnRR ~ extent + (1|study) (*p*‐value of likelihood ratio test <.001). Predictions for low and high extents do not overlap. There was some heteroscedasticity in the model (Figure S6).

**FIGURE 3:**
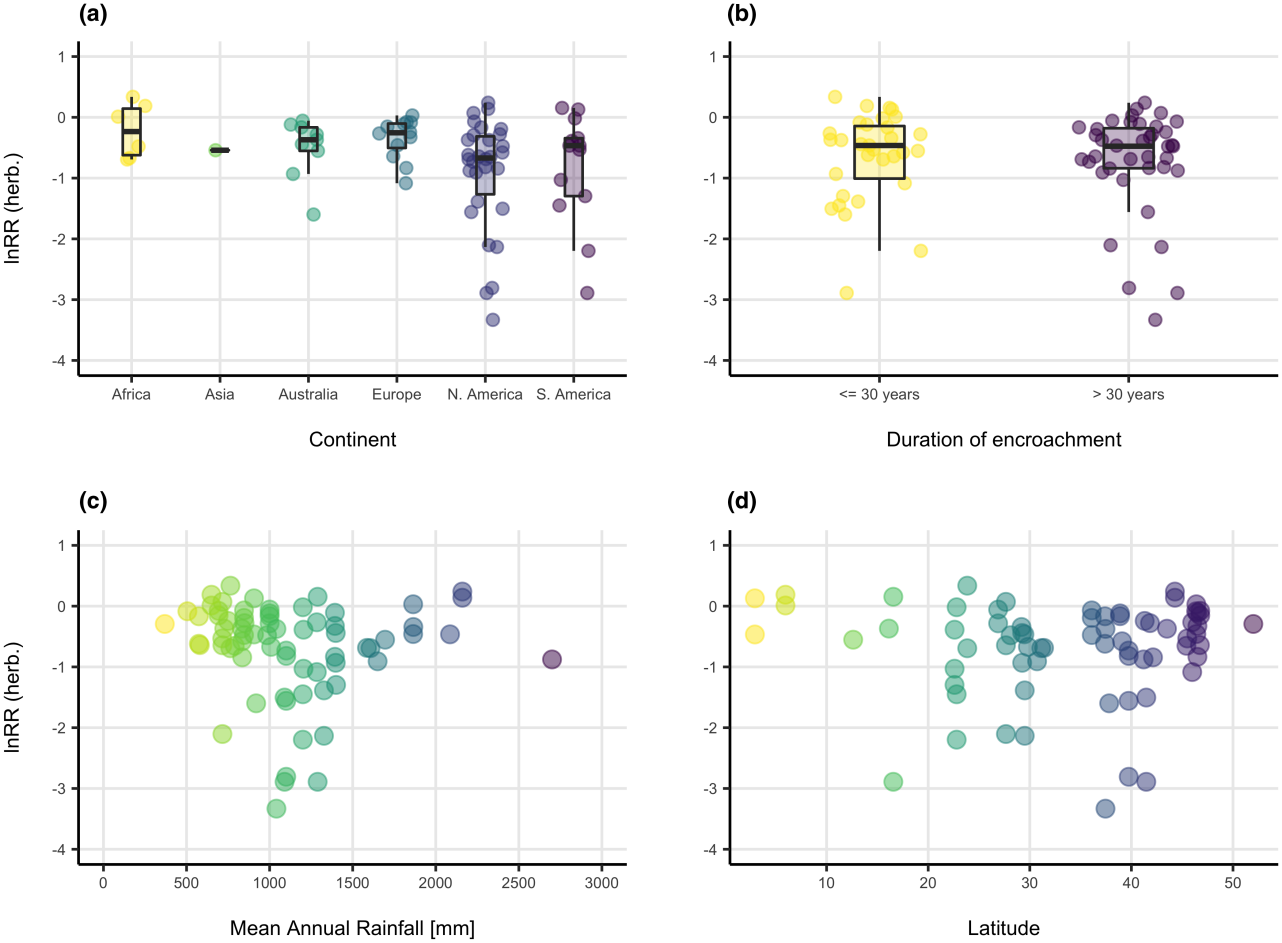
Distribution of lnRR of herbaceous species richness values across (a) continents, (b) categories of encroachment duration, (c) mean annual rainfall gradient, (d) latitude

Within the data there was a clear trend of increasingly negative impact on lnRR of herbaceous species richness with increasing extent of encroachment (Figure 2a). The variance in lnRR values at low extent of encroachment was limited and more varied at medium and high extents, ranging from extremely negative values to slightly positive ones (Figure 2a). In the linear mixed‐effects models testing the relationship of lnRR of herbaceous species richness with extent of encroachment, duration of encroachment, MAR, latitude, continent and the random effect of study, only extent of encroachment had relevance in the data (Table 3) reinforcing the trend observed in the raw data. The univariate model with only extent of encroachment (and the random effect of study) explained 45.4% of variance in lnRR and was the best‐supported model with the lowest AIC (AIC = 161.07). The second most supported model included extent of encroachment and duration of encroachment explaining 45.8% of variance (AIC = 164.59, ΔAIC = 3.52). The initial model including all five fixed effects explained 46.8% of variance (AIC = 195.07, ΔAIC = 34.00). There was no clear dependence of the herbaceous response to encroachment based on continent, duration of encroachment, MAR, or latitude (Figure 3a–d). The null model incorporating only the random effect of study explained 14.5% of variance (AIC = 173.29, ΔAIC = 12.22).

**TABLE 3:** Comparison of models assessing the impact of five fixed effects on the lnRR of herbaceous species richness. Models shown were selected with AIC using backward elimination selection, with the best explanatory power for a model with only one fixed effect (extent of encroachment). All models include a random effect (*study*).

No. of fixed effects	Model	AIC	*R* ^2^
5	lnRR ~ extent + duration + continent + latitude + MAR + (1|study)	195.07	.468
4	lnRR ~ extent + duration + continent + latitude + (1|study)	178.01	.466
3	lnRR ~ extent + duration + continent + (1|study)	169.43	.456
2	lnRR ~ extent + duration + (1|study)	164.59	.458
1	lnRR ~ extent + (1|study)	*161.07*	.454
0	lnRR ~ 1 + (1|study)	173.29	.145

Model predictions indicated a negative lnRR for each extent of encroachment, with predicted lnRR decreasing with the increasing extent (Figure 2b). Only for the low extent of encroachment did the 95% CI span 0 (lnRR = −0.17, 95% CI: −0.49, 0.15). In contrast, the values for medium (lnRR = −0.68, 95% CI: −0.93, −0.43) and high (lnRR = −1.24, 95% CI: −1.56, −0.91) extents of encroachment did not span zero, were substantially negative, and statistically significant. Although the prediction for medium extent of encroachment overlapped with other extents, the predictions for low and high extents of encroachment did not overlap. In 16 out of 17 studies where more than one extent of encroachment was distinguished, lnRR decreased with consecutive categories of increasing extent of encroachment (Figure S7) and reflected the clear overall trend observed by the data and the model (Figure 2a,b). To further check the lack of relevance of duration of encroachment, latitude, continent, and MAR as correlates, comparison of univariate models (one fixed effect and one random effect) with a null model incorporating only the random effect of study confirmed the lack of significance for each (*p*‐values of likelihood ratio tests >.05; Figure S8). Sensitivity test using lnRR > −2 confirmed the highest explanatory power of the extent of encroachment and asserted that medium and high categories of extent were significantly negative (Figure S9).

### 3.2 Global weighted mean response at three extents of encroachment

**FIGURE 4:**
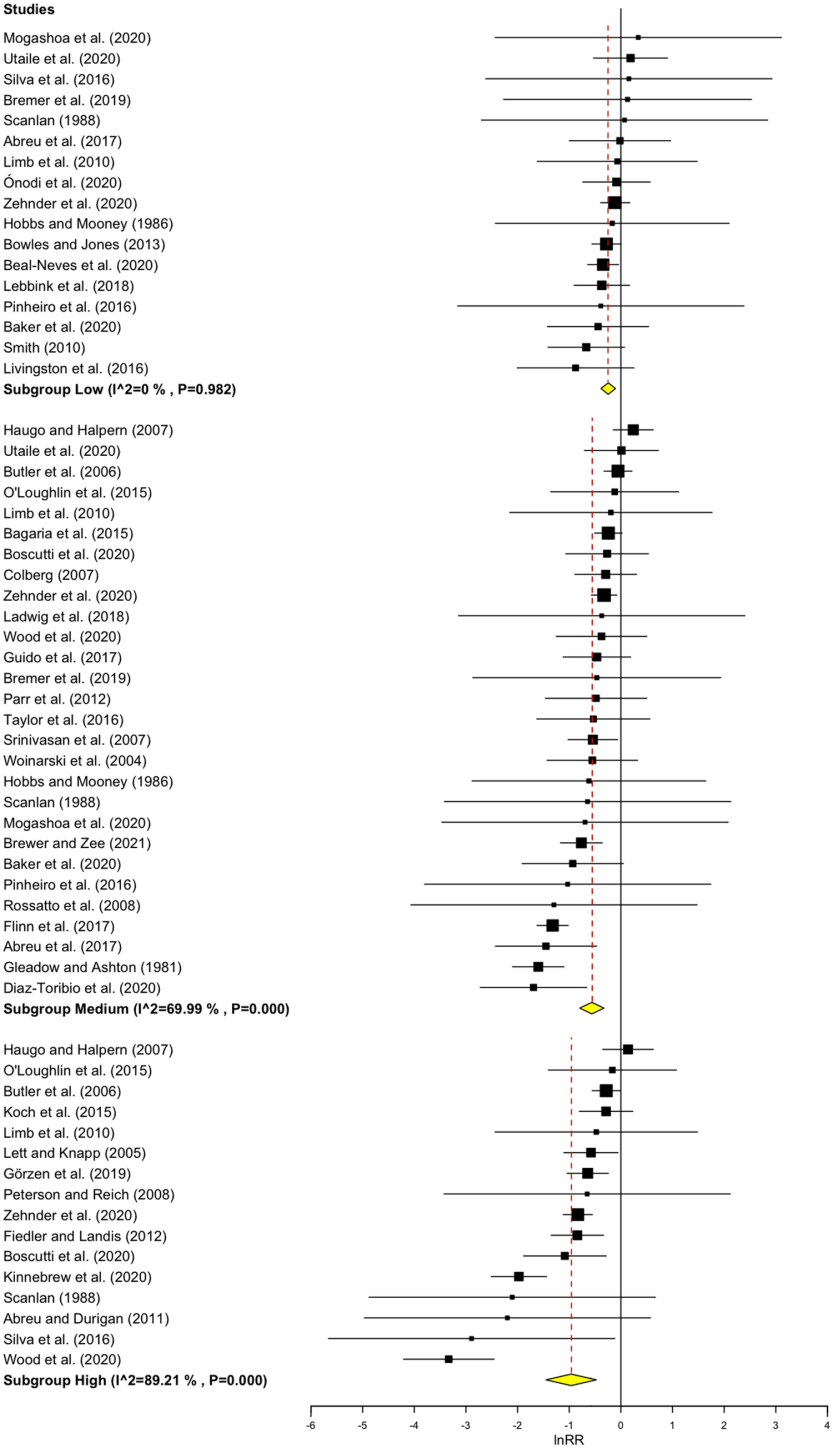
Global weighted mean of herbaceous species richness response to the three extents of encroachment. On the left are listed 61 weighted points of three extent categories from 42 studies (full references in List S2). The studies are ordered by the effect size (from highest to lowest) for each extent category. The size of black squares (effect sizes—lnRR) represents the weighting of each study calculated as the inverted variance. Solid lines represent 95% CIs. Yellow diamonds and red vertical dashed lines represent the weighted mean for each extent category. I^2^ values refer to the dispersion of lnRR—there is low heterogeneity for low extent of encroachment, and high heterogeneity for medium and high categories.

The global weighted mean of lnRR of herbaceous species richness was significantly negative for each extent of woody encroachment, decreasing from −0.245 (95% CI: −0.385, −0.104) at low, to −0.562 (95% CI: −0.796, −0.327) at medium, and to −0.962 (95% CI: −1.448, −0.475) at high extent of encroachment (Figure 4). Only the results for the low extent of encroachment might be uncertain as Rosenberg's fail‐safe number was lower than the cut‐off (Table S3). The sensitivity test of an unweighted model confirmed that the mean lnRR of herbaceous species richness for each extent of encroachment was negative and decreased with increasing extent (Figure S10). However, the 95% CI spans 0 at a low extent.

### 3.3 Functional group responses

**FIGURE 5:**
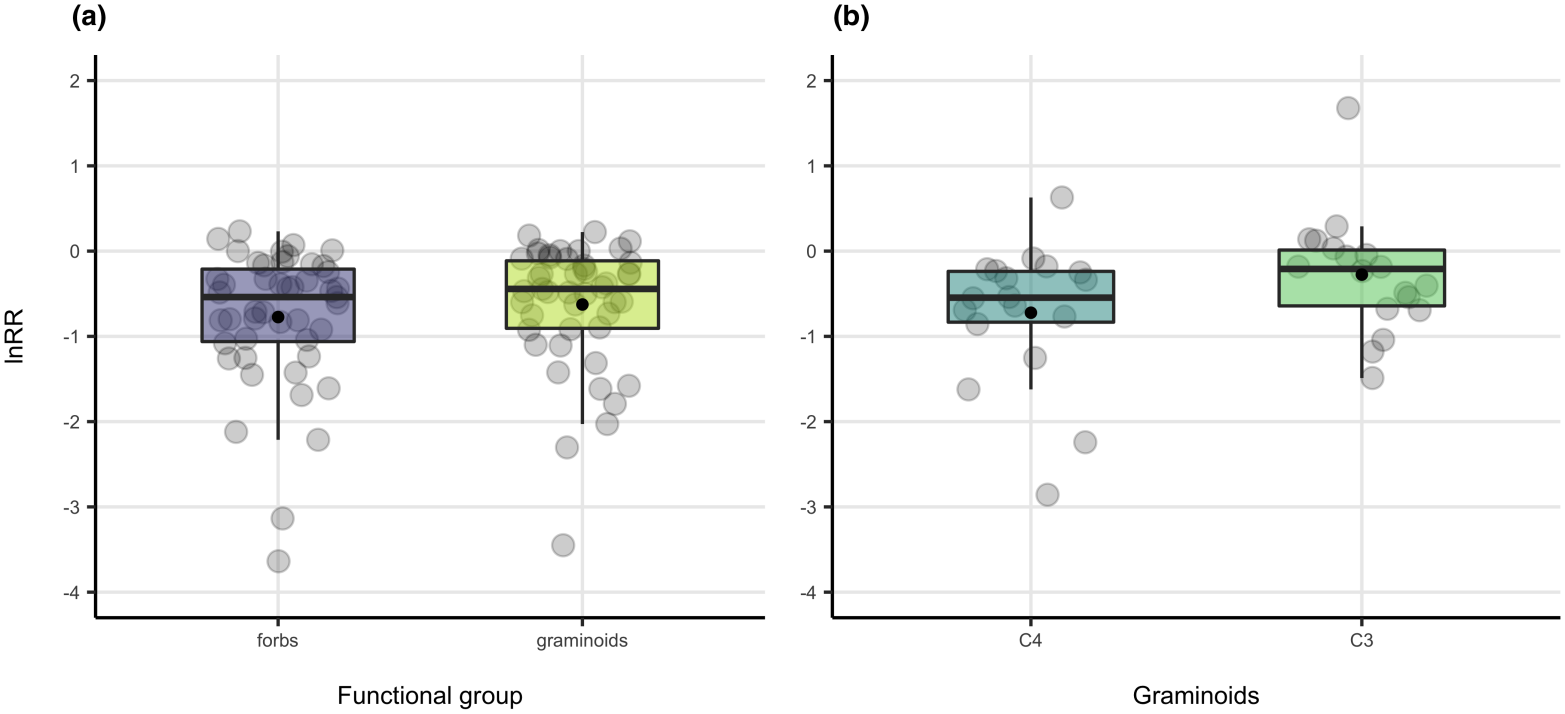
Responses of plant functional groups to encroachment. Comparison of boxplots of (a) lnRR of forb and graminoid species richness and (b) lnRR of C_4_ and C_3_ graminoid species richness. Black dots represent means. Gray dots are individual lnRR values. In (b), studies that only reported totals of C_4_ and C_3_ species were removed because they resulted in extreme lnRR values where very few individuals per group were present.

The lnRR of each of the forb, graminoid, C_3_ graminoid, and C_4_ graminoid functional groups was significantly lower than 0 (Table 2). Pairwise comparisons found that the lnRR of forb species richness was lower than the lnRR of graminoid species richness (mean of 47 differences = −0.148, *p*‐value = .016; Figure 5a), and the lnRR of C_4_ graminoid species richness was lower than the lnRR of C_3_ graminoid species richness (mean of 18 differences = −0.447, *p*‐value = .047; Figure 5b).

## 4 DISCUSSION

Encroachment negatively impacts herbaceous species richness, with the negative impact becoming more severe with increasing extent of encroachment. With the lack of a significant role found for climate and geography in predicting encroachment impacts on herbaceous richness, encroachment should be defined as a globally threatening process in grassy ecosystems. The mean lnRR decreased significantly with increasing extent of encroachment, and with a significant drop in richness between low and high encroachment extents. Based on the weighted mean responses to encroachment, the lnRR of herbaceous species richness decreased from −0.245 at <33% increase in woody cover, to −0.562 at 33%–66%, and to −0.962 at >66%.

The uncertainty of impact was highest at low extent of encroachment. It is unknown whether the combined variability among our analyses and low response values to low extent of encroachment reflect compositional change alongside small declines in richness in heterogenous grassy ecosystems. At a low extent of encroachment, changes in woody cover are potentially patchy and may reflect heterogeneity and the patch dynamics that characterise savannas globally (Scholes & Archer, 1997). The diversity of grassy ecosystems with and without tree cover, i.e. savanna versus grassland, steppe and prairie, likely also plays a role in the variability associated with low levels of encroachment. Grassy ecosystems with heterogeneity in woody cover have gradients in shade and moisture availability (Hoffmann et al., 2012) that enable new plants to colonise, while the woody cover is not extensive enough to exclude grassy ecosystem specialists (Peterson & Reich, 2008). Similarly, sparsely encroached ecosystems have also been viewed as ecotones bearing characteristics of transitional ecosystems that may have relatively higher species richness (Utaile et al., 2020). Given the global trajectory of encroachment, mildly encroached ecosystems are unlikely stable in the absence of active management (Scanlan, 1988; Stevens et al., 2017).

Herbaceous richness significantly declines at medium and high extents of encroachment as local environments become increasingly modified. Impacts on the herbaceous flora range from lowering the overall abundance of herbaceous plants (Fiedler & Landis, 2012), functional turnover and reduced diversity (Pilon et al., 2021), to causing localised extinctions (Abreu & Durigan, 2011). Furthermore, according to the studies compiled, medium to high encroachment changes soil nutrients, often altering the patterns of nitrogen fixation or decreasing pH (Kinnebrew et al., 2020; Zehnder et al., 2020), and persistently decreases ground layer light availability (Taylor et al., 2016). Encroachment can also lead to nutrient deficiencies due to efficient nutrient removal by the encroacher (Baker et al., 2020). Similarly, there are repeated observations of not only increased soil moisture detrimental to native plant diversity (Gleadow & Ashton, 1981; Kinnebrew et al., 2020; Pinheiro et al., 2016) but also water deficiency due to increased competition for water resources and higher evapotranspiration (Abreu & Durigan, 2011; Baker et al., 2020). The increasing extent of encroachment decreases the physical space available aboveground (Wood et al., 2020), and belowground through increased root density (Scanlan, 1988). Encroachment via reducing grass biomass lowers flammability and forage availability in turn altering disturbance regimes and restricting herbivore access (Charles‐Dominique et al., 2018; Koch et al., 2015; Pilon et al., 2021). Medium and high extents of encroachment reflect substantive ongoing changes in the abiotic and biotic environments of ecosystems that culminate in substantive herbaceous species losses.

Surprisingly, none of the geographic or climate variables had relevance in explaining the species richness response to encroachment. Counter‐intuitively, this does provide important insight into encroachment as a process universally detrimental to herbaceous diversity, spanning temperate to tropical grassy ecosystems.

In dryland ecosystems that encompass arid and semi‐arid shrub‐ and grass‐dominated ecosystems, encroachment impacts are variable (Eldridge et al., 2011). Our analyses demonstrate grassy ecosystems characterised by a wide range of rainfall, including those where MAR > 1100 mm that have been excluded from previous meta‐analyses, are negatively affected by encroachment. Grassy ecosystems and drylands can be confused in the literature despite their functional differences, and this underlines the necessity to adequately represent encroachment effects in grassy ecosystems. Although lnRR was consistently negative at the entire MAR range, interestingly, the lowest lnRR values (<−1) were found in the MAR range of 716–1400 mm (Figure 3c). One potential explanation can be the bi‐stability of ecosystems where e.g. tropical savannas can abruptly change to a closed‐canopy ecosystem through disturbance‐exclusion resulting in significant biodiversity change (Bond & Parr, 2010). However, some low values in this range represent temperate grasslands (e.g. Flinn et al., 2017). It is unclear from these analyses whether bi‐stability plays a role and should be a priority for future work if thresholds of resilience are to be determined.

Despite different search criteria from Ratajczak et al. (2012), the average response of total plant species richness for North America from that study (lnRR = −0.65) is similar to the global average we observe for herbaceous richness (lnRR = −0.69 ± 0.09). Ratajczak et al. (2012) suggested the negative impact of encroachment observed in North America was distinct from Eldridge et al. (2011) as a product of regional positive and negative patterns. Our data show that when assessing only herbaceous plants in grassy ecosystems, the trend is for decreasing species richness across varied geographies, without any distinct regional increases in richness. We also note the unequal geographies of research (and research investment) that produced the uneven distribution of study sites and durations of encroachment observed. Even though encroachment is widespread across Asia and Africa (Kumar et al., 2020; Stevens et al., 2017), only one and four studies respectively were identified there. Less‐studied regions require urgent attention as it appears all grassy ecosystems are likely negatively impacted with consequences for the unique biodiversity of each region.

Although duration of encroachment had little relevance in these data that does not mean duration of encroachment has no impact, rather the response can be variable and ecosystem‐specific. With ongoing encroachment, the balance of herbaceous richness depends on colonisation and extinction, where both local and landscape‐level factors determine their magnitude and chronology (Bagaria et al., 2015; Jackson & Sax, 2010; Zulka et al., 2014). Abreu and Durigan (2011) observed no colonisations and an almost immediate extinction of most species, as 92% of species were shade‐intolerant. On the other hand, Haugo and Halpern (2007) reported relatively quick colonisation by a suite of shade‐tolerant species (with close proximity to a source population), while the extinction of grassland specialists was slower. The duration of encroachment can have different impacts determined by (1) the rate at which biodiversity is deprived of favorable conditions—depending on the extent of encroachment and species' tolerance of change, and (2) how quickly (and whether at all) new species can immigrate—depending on dispersal capability and proximity of seed sources (Bagaria et al., 2015). Potentially, if only grassy ecosystem specialists were counted in our meta‐analysis, a trend related to time would be detected.

Encroachment leads to the replacement of forbs and graminoids by litter, shrubs, or woody vines (Pinheiro et al., 2016). Both forbs and graminoids were negatively impacted by encroachment, with forbs experiencing a slight but still significantly larger decline in richness compared with graminoids. Forbs are a species‐rich and taxonomically diverse functional group in grassy ecosystems, with species potentially more geographically restricted than grasses in their ranges (Bond & Parr, 2010). In fire‐maintained ecosystems, many forb species have large underground storage organs and flower post‐fire (Bond, 2016). Consequently, where encroachment is a result of fire suppression, declines in forb species richness are commonly observed (Parr et al., 2012; Peterson & Reich, 2008). Both C_3_ and C_4_ graminoids negatively respond to encroachment, however, C_3_ graminoids relatively less so than C_4_ graminoids. C_4_ graminoids typify and define tropical grassy ecosystems, and generally have high light requirements due to their morpho‐physiology, and therefore are often reduced in richness or completely lost from encroached ecosystems where disturbance is infrequent (Diaz‐Toribio et al., 2020; Peterson & Reich, 2008; Wood et al., 2020). Beyond our observations for functional groups, woody encroachment likely leads to habitat homogenisation and predominance of a single or only a few herbaceous species (Mogashoa et al., 2020; Pinheiro et al., 2016). Moreover, rare and threatened species can decline in abundance and go locally extinct (Costello et al., 2000; Pinheiro et al., 2016), while ruderals and non‐native species can increase in numbers (Lett & Knapp, 2005).

Grassy ecosystems require active management to mitigate, halt and reverse encroachment. Considering that long‐lasting environmental changes, such as altered soil chemistry, can be a product of encroachment and barrier to restoration (Kinnebrew et al., 2020), it would be most effective to prevent or mitigate encroachment in its early stages. In fire‐adapted ecosystems, there is a clear need for appropriate fire regimes to promote plant diversity and heterogeneity within ecosystems (Abreu et al., 2017). In some studies, long‐term declines in grazer numbers contributed to encroachment but grazing reintroductions alone are often insufficient for restoration due to reduced forage quality and changed habitat structures (Zehnder et al., 2020). While mechanical removal or mowing can be implemented, it is resource‐intensive and expensive, restricting the capacity for application (Kinnebrew et al., 2020).

Beyond local management needs to mitigate encroachment, there is an important role for appropriate land policies recognising the importance of land stewardship and unique ecology of grassy ecosystems. Over the last centuries, in addition to increasing p[CO_2_] and climate change, grassy ecosystems have been severely impacted by changed patterns of human use. Across regions, dispossession of Indigenous peoples who had stewarded lands for millennia and resultant shifts in land tenure and use led to the placement of fences and roads in landscapes, fire suppression, fragmentation, decline and extinction of animal populations, and displacement of wildlife with livestock (Costello et al., 2000; Laris, 2002; Stevens et al., 2017). There is now a tendency to implement programs advocating the addition of trees as a nature‐based solution to climate change that can be often ignorant of existing carbon stored in intact grassy ecosystem soils and vegetation and how this may impact biodiversity (Zhou et al., 2022). Ensuring sustainability of grassy ecosystem biodiversity worldwide requires ecologically and socio‐culturally appropriate policies to mitigate continued p[CO_2_] rises (that reinforce encroachment) and encompass centennial‐scale views on ecosystem integrity.

## 5 CONCLUSION

With encroachment occurring globally, herbaceous species richness is widely declining, with impacts becoming increasingly severe with increasing extent of encroachment. The trend of reduced species richness with encroachment is not directly related to geography and climate, highlighting encroachment as an emergent property of global change driven by a multitude of factors with the same result of declining herbaceous diversity. With encroachment, richness of forbs, C_3_ and C_4_ graminoids is declining, with the ongoing disappearance of grassy ecosystem specialists. Those losses are rarely compensated for with increases in species suited to the new environmental conditions. Given the importance of extent of encroachment in predicting lnRR, research is urgently required to understand the thresholds of woody cover that equate to resilient and biodiverse grassy ecosystems; the compositional change in the flora and how this may be mirrored in faunal changes; and the traits of encroaching species with regards to the impact on biodiversity, as the effects of encroachment have been shown to differ among species (Zehnder et al., 2020). Finally, we highlight the need for environmental policy and management centred on grassy biomes to preserve their natural biodiversity and ecosystem integrity as there are no grassy biomes without grasses.

## AUTHOR CONTRIBUTIONS

Jakub D. Wieczorkowski and Caroline E. R. Lehmann designed research; Jakub D. Wieczorkowski compiled and analysed data; Jakub D. Wieczorkowski and Caroline E. R. Lehmann wrote the paper.

## CONFLICT OF INTEREST

The authors have declared no conflict of interest.

## Supporting information


Appendix S1

## Data Availability

The data that support the findings of this study, along with R code used for analyses, are available on Figshare at https://doi.org/10.6084/m9.figshare.19982180 (Wieczorkowski & Lehmann, 2022).
