# Supplementary material for: Encroachment diminishes herbaceous plant diversity in grassy ecosystems worldwide
Source: Glob Chang Biol. 2022 Jul 11;28(18):5532–46. doi: 10.1111/gcb.16300 (PMC9544121; doi:10.1111/gcb.16300)
Supplement: Supplementary file 1 — Appendix S1 [file GCB-28-5532-s001.docx]

Supplementary Information: Lists (S1-S2) of exclusion criteria and studies used in the meta-analysis; and Tables (S1-S3) and Figures (S1-S10) with results of assumption checks, sensitivity tests and a test for publication bias

**Encroachment diminishes herbaceous plant diversity in grassy ecosystems worldwide**

Jakub D. Wieczorkowski*^1,2^ and Caroline E.R. Lehmann^1,2^

^1^ School of GeoSciences, The University of Edinburgh, Edinburgh EH9 3FF, United Kingdom

^2^ Tropical Diversity, Royal Botanic Garden Edinburgh, Edinburgh EH3 5LR, United Kingdom

*Corresponding Author: [jakub.wieczorkowski@ed.ac.uk](mailto:jakub.wieczorkowski@ed.ac.uk)

Contents

**List S1:** List of exclusion criteria used in study selection.

**List S2:** List of studies used in the meta-analysis.

**Table S1**: Collinearity of five fixed effects included in linear mixed-effects model analyses.

**Table S2**: Comparison of lnRR data distribution from Web of Science search vs full search.

**Table S3**: Fail-safe N test for meta-analysis in OpenMEE.

**Figure S1**: Correlation of latitude and Mean Annual Temperature.

**Figure S2**: Paired correlations among the five fixed effects.

**Figure S3**: Histograms of all modelled response variables.

**Figure S4**: Normal QQ plots of modelled response variables.

**Figure S5**: The effect of method of richness calculation, plot size, and cause of encroachment on lnRR (herb.).

**Figure S6**: Homoscedasticity check of the univariate model with the extent of encroachment and random effect of study.

**Figure S7**: Visualisation of lnRR (herb.) for studies with more than one extent of encroachment.

**Figure S8**: Predictions of lnRR (herb.) from univariate models with the four remaining fixed effects.

**Figure S9**: Sensitivity test: examining correlates of herbaceous richness response to encroachment for lnRR > -2.

**Figure S10**: Sensitivity test: global (un)weighted mean of herbaceous species richness response to the three extents of encroachment.

#### **List S1.** List of exclusion criteria used in study selection.

When examining encroachment impacts, there are a series of confounding factors which can influence ecosystem response to changes to encroachment. We had to be careful in distinguishing the impact of encroachment alone as much as possible. Consequently, studies were excluded for the following criteria:

1. fertiliser use;
2. ploughing or past tillage for agriculture, plantation, regular mowing, woody plant removal, intensive land use such as cattle farms, or any other use which would cause the unencroached ecosystem to be significantly degraded;
3. studies primarily designed to detect impacts of time since fire (if less than 15 years);
4. comparisons that centred on differences in plant diversity before and after a single fire to avoid overrepresentation of fire effects rather than encroachment;
5. ecosystems restored with fire after long-term encroachment as we expect patterns of species richness in re-opened ecosystems would be at odds with those undergoing encroachment;
6. encroachment was simultaneous with exotic grass invasion, and consequently it was unclear what changes in diversity can be attributed to encroachment;
7. grazing regimes substantially different over time or space between the encroached and unencroached ecosystems (although natural variation in animal use of sites was accepted, e.g. due to increasing difficulty in access). We decided to implement this criterion because in cases where encroached and unencroached areas have different grazing intensities, encroachment effects are often indistinguishable from grazing effects which can already be highly diverse themselves. Given the already diverse range of studies from ecosystems worldwide which implement different methodologies, the criterion allowed for higher comparability in detecting the effect of encroachment.

**List S2**. List of studies used in the meta-analysis.

Abreu, R. C. R., & Durigan, G. (2011). Changes in the plant community of a Brazilian grassland savannah after 22 years of invasion by *Pinus elliottii* Engelm. *Plant Ecology and Diversity,* 4(2-3), 269–278. doi.org/10.1080/17550874.2011.594101

Abreu, R. C. R., Hoffmann, W. A., Vasconcelos, H. L., Pilon, N. A., Rossatto, D. R., & Durigan, G. (2017). The biodiversity cost of carbon sequestration in tropical savanna. *Science Advances*, 3(8), e1701284. doi.org/10.1126/sciadv.1701284

Bagaria, G., Helm, A., Rodà, F., & Pino, J. (2015). Assessing coexisting plant extinction debt and colonization credit in a grassland–forest change gradient. *Oecologia*, 179, 823–834. doi.org/10.1007/s00442-015-3377-4

Baker, A. G., Catterall, C., Benkendorff, K., & Fensham, R. J. (2020). Rainforest expansion reduces understorey plant diversity and density in open forest of eastern Australia. *Austral Ecology*, 45(5), 557–571. doi.org/10.1111/aec.12871

Beal-Neves, M., Chiarani, E., Ferreira, P. M. A., & Fontana, C. S. (2020). The role of fire disturbance on habitat structure and bird communities in South Brazilian Highland Grasslands. *Scientific Reports*, 10, 19708. doi.org/10.1038/s41598-020-76758-z

Boscutti, F., Pellegrini, E., Casolo, V., de Nobili, M., Buccheri, M., & Alberti, G. (2020). Cascading effects from plant to soil elucidate how the invasive *Amorpha fruticosa* L. impacts dry grasslands. *Journal of Vegetation Science*, 31, 667–677. doi.org/10.1111/jvs.12879

Bowles, M. L., & Jones, M. D. (2013). Repeated burning of eastern tallgrass prairie increases richness and diversity, stabilizing late successional vegetation. *Ecological Applications*, 23, 464–478. doi.org/10.1890/12-0808.1

Bremer, L. L., Farley, K. A., DeMaagd, N., Suárez, E., Cárate Tandalla, D., Vasco Tapia, S., & Mena Vásconez, P. (2019). Biodiversity outcomes of payment for ecosystem services: lessons from páramo grasslands. *Biodiversity and Conservation*, 28, 885–908. doi.org/10.1007/s10531-019-01700-3

Brewer, J. S., & Zee, P. (2021). Functional diversity and coexistence of herbaceous plants in wet, species-rich savannas. *Ecology and Evolution*, 11, 5111–5120. doi.org/10.1002/ece3.7404

Butler, D. W., Fairfax, R. J., & Fensham, R. J. (2006). Impacts of tree invasion on floristic composition of subtropical grasslands on the Bunya Mountains, Australia. *Australian Journal of Botany*, 54(3), 261–270. doi.org/10.1071/BT05070

​​Colberg, T. J. (2007). *Relationships between plant communities and soil carbon in the prairie ecozone of Saskatchewan*. PhD Thesis. University of Saskatchewan.

Diaz-Toribio, M. H., Carr, S., & Putz, F. E. (2020). Pine savanna plant community disassembly after fire suppression. *Journal of Vegetation Science*, 31, 245–254. doi.org/10.1111/jvs.12843

Fiedler, A. K., & Landis, D. A. (2012). Biotic and Abiotic Conditions in Michigan Prairie Fen Invaded by Glossy Buckthorn (*Frangula alnus*). *Natural Areas Journal*, 32(1), 41–53. doi.org/10.3375/043.032.0106

Flinn, K. M., Mikes, J. L., & Kuhns, H. A. D. (2017). Plant diversity and community composition in eastern North American serpentine barrens. *Journal of the Torrey Botanical Society*, 144, 125–138. doi.org/10.3159/torrey-d-16-00030

Gleadow, R. M., & Ashton, D. H. (1981). Invasion by *Pittosporum undulatum* of the Forests of Central Victoria. I. Invasion patterns and plant morphology. *Australian Journal of Botany*, 29(6), 705–720. doi.org/10.1071/BT9810705

Görzen, E., Borisova, K., Fenesi, A., Ruprecht, E., & Donath, T. W. (2019). Effects of woody species encroachment and fire on vegetation and the soil seed bank in dry grasslands of Transylvania. *Applied Vegetation Science*, 22, 409–422. doi.org/10.1111/avsc.12435

Guido, A., Salengue, E., & Dresseno, A. (2017). Effect of shrub encroachment on vegetation communities in Brazilian forest-grassland mosaics. *Perspectives in Ecology and Conservation*, 15(1), 52–55. doi.org/10.1016/j.pecon.2016.11.002

Haugo, R. D., & Halpern, C. B. (2007). Vegetation responses to conifer encroachment in a western Cascade meadow: A chronosequence approach. *Canadian Journal of Botany*, 85, 285–298. doi.org/10.1139/B07-024

Hobbs, R. J., & Mooney, H. A. (1986). Community changes following shrub invasion of grassland. *Oecologia*, 70, 508–513. doi.org/10.1007/BF00379896

Kinnebrew, E., Champlin, L. K., Galford, G. L., & Neill, C. (2020). Woody plant encroachment into coastal grasslands: consequences for soil properties and plant diversity. *Regional Environmental Change*, 20, 94. doi.org/10.1007/s10113-020-01687-6

Koch, B., Edwards, P. J., Blanckenhorn, W. U., Walter, T., & Hofer, G. (2015). Shrub encroachment affects the diversity of plants, butterflies, and grasshoppers on two Swiss subalpine pastures. *Arctic, Antarctic, and Alpine Research*, 47(2), 345–357. doi.org/10.1657/AAAR0013-093

Ladwig, L. M., Damschen, E. I., & Rogers, D. A. (2018). Sixty years of community change in the prairie-savanna-forest mosaic of Wisconsin. *International Journal of Business Innovation and Research*, 8(16), 8458–8466. doi.org/10.1002/ece3.4251

Lebbink, G., Fensham, R., & Cowley, R. (2018). Vegetation responses to fire history and soil properties in grazed semi-arid tropical savanna. *Rangeland Journal*, 40(3), 271–285. doi.org/10.1071/RJ17075

Lett, M. S., & Knapp, A. K. (2005). Woody plant encroachment and removal in mesic grassland: Production and composition responses of herbaceous vegetation. *American Midland Naturalist*, 153(2), 217–231. doi.org/10.1674/0003-0031(2005)153[0217:WPEARI]2.0.CO;2

Limb, R. F., Engle, D. M., Alford, A. L., & Hellgren, E. C. (2010). Tallgrass prairie plant community dynamics along a canopy cover gradient of eastern redcedar (*Juniperus virginiana* L.). *Rangeland Ecology and Management*, 63(6), 638–644. doi.org/10.2111/REM-D-09-00056.1

Livingston, A. C., Varner, J. M., Jules, E. S., Kane, J. M., & Arguello, L. A. (2016). Prescribed fire and conifer removal promote positive understorey vegetation responses in oak woodlands’ *Journal of Applied Ecology*, 53(5), 1604–1612. doi.org/10.1111/1365-2664.12703

Mogashoa, R., Dlamini, P., & Gxasheka, M. (2020). Grass species richness decreases along a woody plant encroachment gradient in a semi-arid savanna grassland, South Africa. *Landscape Ecology*, 36(2), 617–636. doi.org/10.1007/s10980-020-01150-1

O’Loughlin, L. S., Green, P. T., & Morgan, J. W. (2015). The rise and fall of *Leptospermum laevigatum*: plant community change associated with the invasion and senescence of a range-expanding native species. *Applied Vegetation Science*, 18(2), 323–331. doi.org/10.1111/avsc.12131

​​Ónodi, G., Kertész, M., Lengyel, A., Pándi, I., Somay, L., Szitár, K., & Kröel-Dulay, G. (2021). The effects of woody plant encroachment and wildfire on plant species richness and composition: Temporal changes in a forest-steppe mosaic. *Applied Vegetation Science*, 24(1), e12546. doi.org/10.1111/avsc.12546

Parr, C. L., Gray, E. F., & Bond, W. J. (2012). Cascading biodiversity and functional consequences of a global change-induced biome switch. *Diversity and Distributions*, 18(5), 493–503. doi.org/10.1111/j.1472-4642.2012.00882.x

Peterson, D. W., & Reich, P. B. (2008). Fire frequency and tree canopy structure influence plant species diversity in a forest-grassland ecotone. *Plant Ecology*, 194, 5–16. doi.org/10.1007/s11258-007-9270-4

​​Pinheiro, L. F. S., Kolb, R. M., & Rossatto, D. R. (2016). Changes in irradiance and soil properties explain why typical non-arboreal savanna species disappear under tree encroachment. *Australian Journal of Botany*, 64(4), 333–341. doi.org/10.1071/BT15283

Rossatto, D. R., Toniato, M. T. Z., & Durigan, G. (2008). Flora fanerogâmica não-arbórea do cerrado na Estação Ecológica de Assis, Estado de São Paulo. *Revista Brasileira de Botanica*, 31(3), 409–424. doi.org/10.1590/S0100-84042008000300005

Scanlan, J. C. (1988). *Spatial and temporal vegetation patterns in a subtropical Prosopis savanna woodland, Texas*. PhD Dissertation. Texas A&M University.

Silva, F. H. B, Arieira, J., Parolin, P., Nunes da Cunha, C., & Junk, W. J. (2016). Shrub encroachment influences herbaceous communities in flooded grasslands of a neotropical savanna wetland. *Applied Vegetation Science*, 19(3), 391–400. doi.org/10.1111/avsc.12230

Smith, F. R. (2010). Using plant functional types to compare vegetation structure of alien-invaded and uninvaded *Acacia nilotica* savannas. *South African Journal of Botany*, 76(2), 365–368. doi.org/10.1016/j.sajb.2009.12.004

Srinivasan, M. P., Shenoy, K., & Gleeson, S. K. (2007). Population structure of Scotch broom (*Cytisus scoparius*) and its invasion impacts on the resident plant community in the grasslands of Nilgiris, India. *Current Science*, 93(8), 1108–1113.

Taylor, K. T., Maxwell, B. D., Pauchard, A., Nuñez, M. A., & Rew, L. J. (2016). Native versus non-native invasions: Similarities and differences in the biodiversity impacts of *Pinus contorta* in introduced and native ranges. *Diversity and Distributions*, 22(5), 578–588. doi.org/10.1111/ddi.12419

Utaile, Y. U., Honnay, O., Muys, B., Cheche, S. S., & Helsen, K. (2020). Effect of *Dichrostachys cinerea* encroachment on plant species diversity, functional traits and litter decomposition in an East-African savannah ecosystem. *Journal of Vegetation Science*, 32(1), e12949. doi.org/10.1111/jvs.12949

Woinarski, J. C. Z., Risler, J., & Kean, L. (2004). Response of vegetation and vertebrate fauna to 23 years of fire exclusion in a tropical *Eucalyptus* open forest, Northern Territory, Australia. *Austral Ecology*, 29(2), 156–176. doi.org/10.1111/j.1442-9993.2004.01333.x

Wood, L. K., Hays, S., & Zinnert, J. C. (2020). Decreased temperature variance associated with biotic composition enhances coastal shrub encroachment. *Scientific Reports*, 10, 8210. doi.org/10.1038/s41598-020-65161-3

Zehnder, T., Lüscher, A., Ritzmann, C., Pauler, C. M., Berard, J., Kreuzer, M., & Schneider, M. K. (2020). Dominant shrub species are a strong predictor of plant species diversity along subalpine pasture-shrub transects. *Alpine Botany*, 130, 141–156. doi.org/10.1007/s00035-020-00241-8

| **Variable** | **GVIF** | **Df** | **GVIF^(1/(2*Df))** |
| --- | --- | --- | --- |
| **extent** | 1.12 | 2 | 1.03 |
| **duration** | 1.26 | 1 | 1.12 |
| **MAR** | 1.10 | 1 | 1.05 |
| **latitude** | 2.37 | 1 | 1.54 |
| **continent** | 2.88 | 4 | 1.14 |

#### **Table S1**. Collinearity of five fixed effects included in linear mixed-effects model analyses. The variables are not correlated. GVIF – generalised variance-inflation factors were calculated because some variables are categorical and have more than 1 degree of freedom (Df) (Fox and Monette, 1992). GVIF^(1/(2*Df)) is the adjustment for the dimension of the confidence ellipsoid. All values are close to 1, while values of 5-10 would indicate significant collinearity which should be further assessed (James et al., 2014).

|  | **lnRR – WOS only** | **lnRR - Full** |
| --- | --- | --- |
| **Min** – **Max** | -3.33 – 0.34 | -3.33 – 0.34 |
| **% negative** | 87% | 87% |
| **Median** | -0.466 | -0.475 |
| **Mean** | -0.671 | -0.689 |
| **n** | 63 | 71 |

#### **Table S2**. Comparison of lnRR data distribution from Web of Science search vs full search. The values of lnRR of herbaceous species richness from the Web of Science (WOS) search are highly similar to the full dataset used (i.e. including the five extra studies). The range and the percentage of negative values are the same. With a precision of 0.001, the mean is 0.018 higher and the median is 0.009 higher for WOS than for the full search.

| **Category of extent** | **Number of studies (n)** | **Minimum cut-off [5*n + 10]** | **Fail-safe number** |
| --- | --- | --- | --- |
| Low | 17 | 95 | 35 |
| Medium | 28 | 150 | 579 |
| High | 16 | 90 | 407 |

#### **Table S3**. Fail-safe N test for meta-analysis in OpenMEE was conducted using fsn() function in package metafor (Viechtbauer, 2010). It was used to understand how many studies with the same weight as the average value would be needed to make the results non-significant. Fail-safe number was greater than the minimum cut-off for medium and high categories of extent of encroachment. For the low extent of encroachment, the fail-safe number was below the minimum cut-off (35 < 95).

**
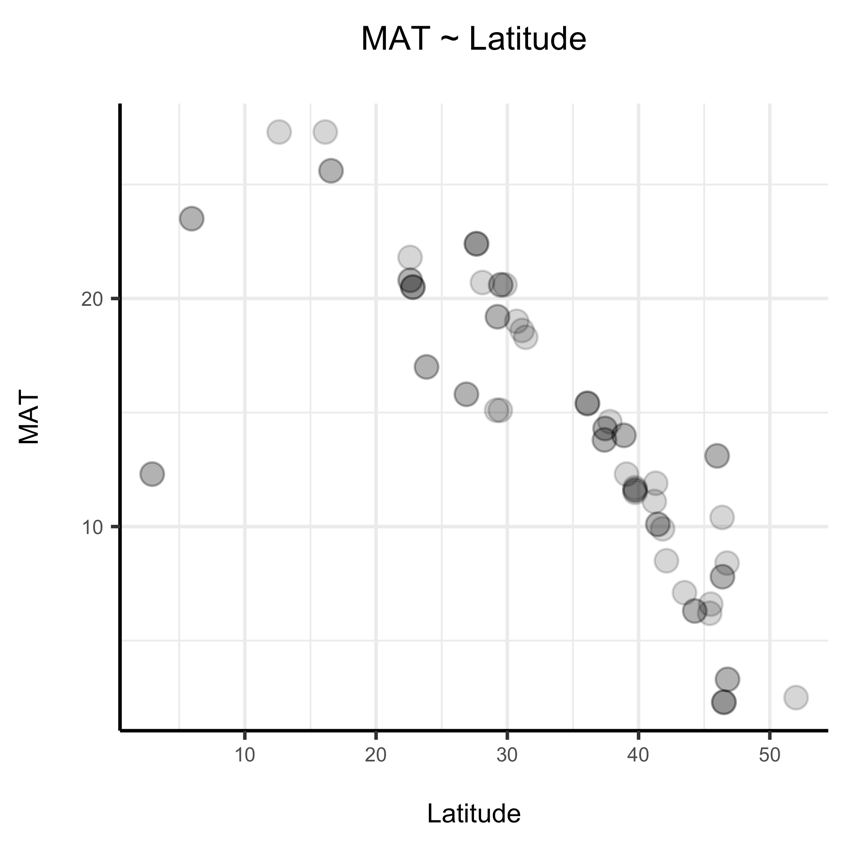
**

#### **Figure S1.** Correlation of latitude and Mean Annual Temperature (MAT). The two variables are highly correlated (Pearson’s r = -0.78, 95%CI: -0.86, -0.67; p-value < 0.001) and therefore we decided that instead of introducing MAT as a separate variable, latitude can be treated as a proxy for MAT in our analyses.

#### **
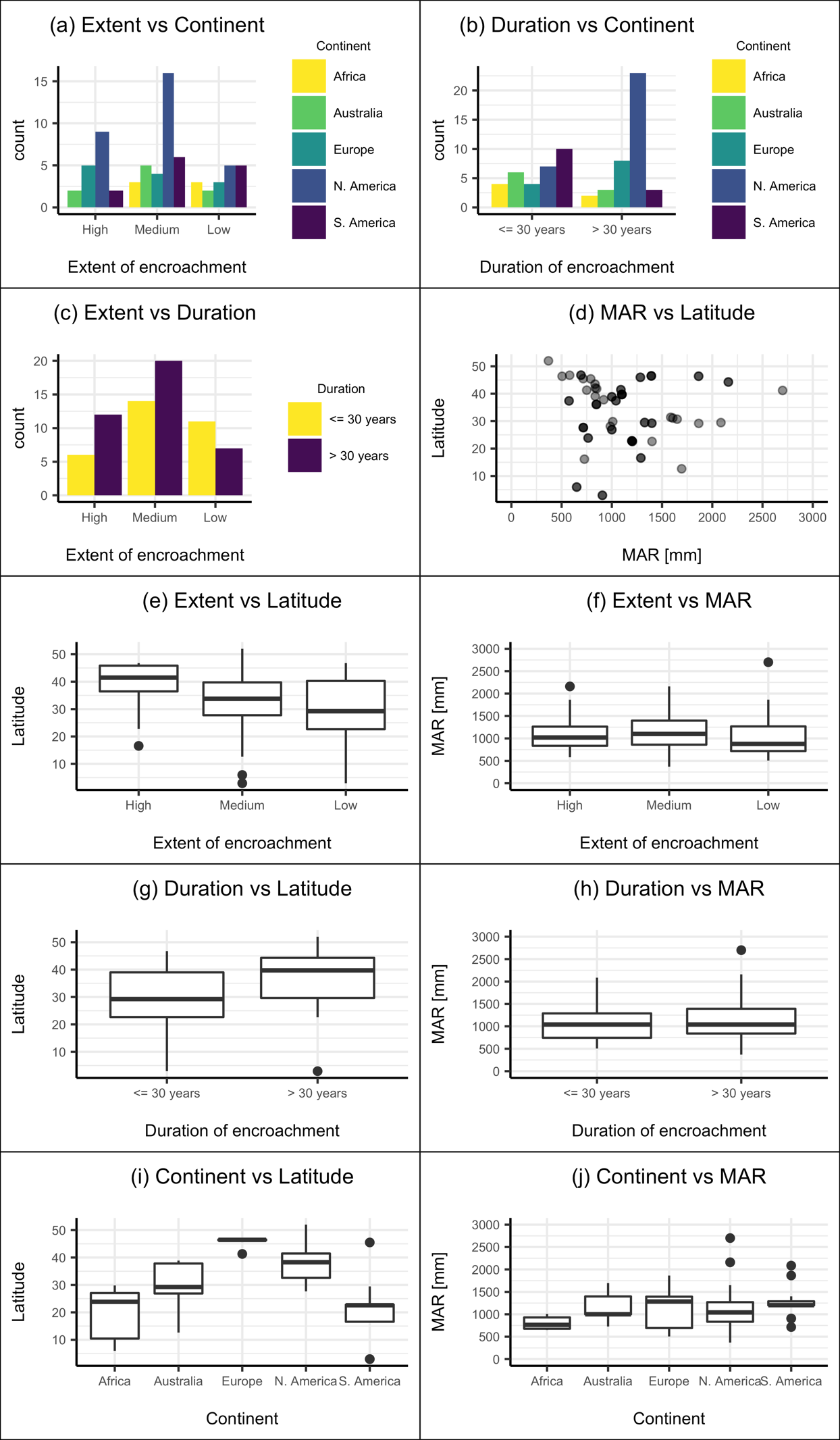
**

#### **Figure S2**. Paired correlations among the five fixed effects. Chi-square tests were conducted for pairs of categorical variables (a-c), Pearson’s correlation coefficient was calculated for a pair of continuous variables (d), and ANOVAs were conducted for pairs of continuous and categorical variables (e-j). P-values associated with each test: (a) ​​0.479; (b) 0.006**; (c) 0.215; (d) 0.652, Pearson’s r = 0.055, 95%CI: -0.182, 0.286; (e) 0.0497*; (f) 0.736; (g) 0.007**; (h) 0.343; (i) < 0.001***; (j) 0.355. It was expected that some tests would surpass the 0.05 significance level considering the high numbers of levels in categorical variables and generally low sample sizes. The correlations at (b), (e), and (g) are likely detected by chance and are not theoretically important. Therefore, they do not have to be removed from the analyses. Moreover, both variables from each pair represent distinct issues and the two cannot be treated as proxies. In (b) and (g), longer duration of encroachment found at higher latitudes and continents of North America and Europe are possibly because long-term records of encroachment have been recorded in countries with more funding opportunities for such studies and long-term research focus. In (e), more records of the high category of the extent of encroachment at high latitudes are rather due to the types of studies conducted and not because of any characteristic of encroachment (extent of encroachment is the relative change in woody cover recorded in the study, not a maximum woody cover possible in a given area). In (i), the correlation of continent and latitude is significant and expected, however, we decided to still use both variables because they can influence different aspects of diversity response to encroachment – for example, lnRR patterns could differ across continents as suggested by Ratajczak et al. (2012); or possibly lnRR patterns could differ across low latitudes (tropics) and high latitudes (temperate areas). Moreover, continent and latitude were not collinear enough in the initial model to be removed (see Table S1).

#### **
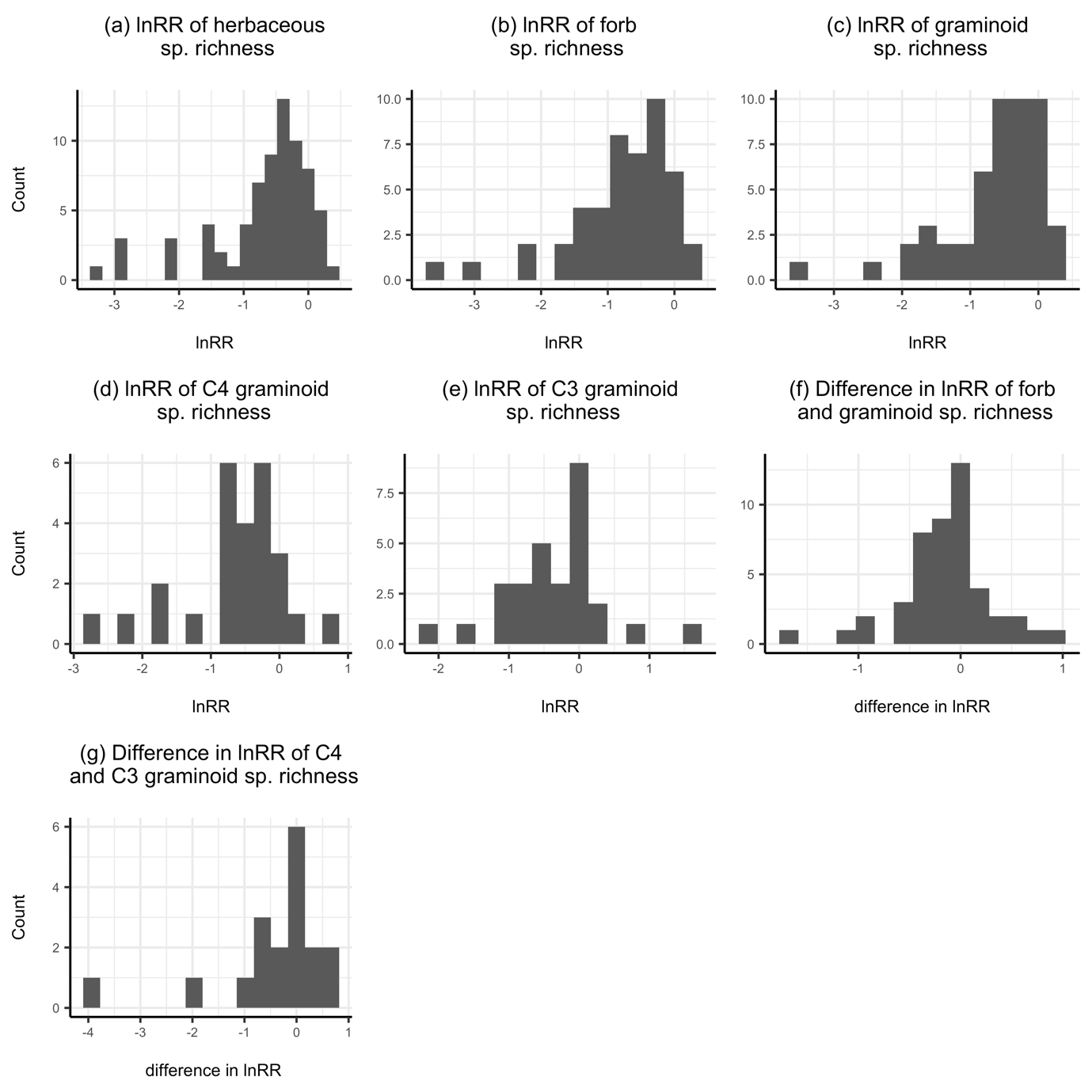
**

#### **Figure S3**. Histograms of all modelled response variables (i.e. lnRR values or differences in lnRR for functional group comparisons). (a), (b), (c) and (g) seem to be slightly negatively skewed, while normal distribution can be assumed for (d), (e), (f).

| (a) lnRR of herbaceous sp. richness | (b) lnRR of forb sp. richness | (c) lnRR of graminoid sp. richness |
| --- | --- | --- |
| 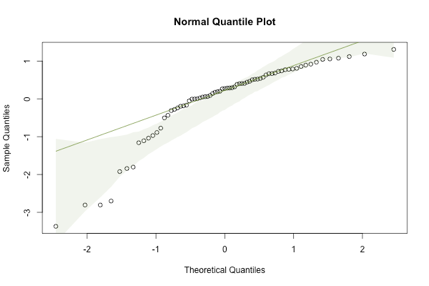 | 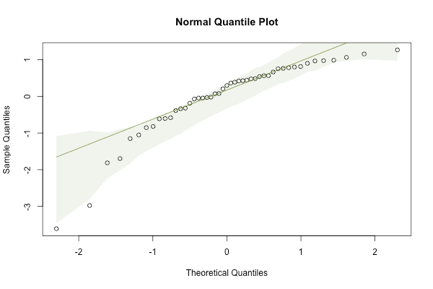 | 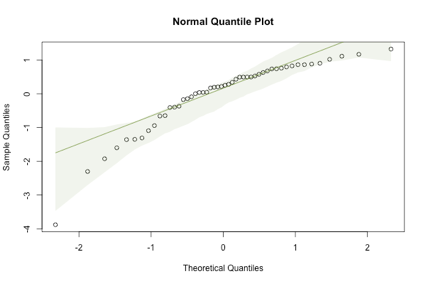 |
| (d) lnRR of C4 graminoid sp. richness | (e) lnRR of C3 graminoid sp. richness | (f) difference in lnRR (forb vs graminoid) |
| 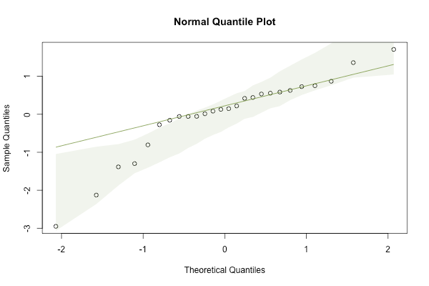 | 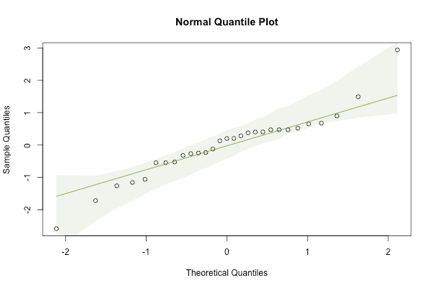 | 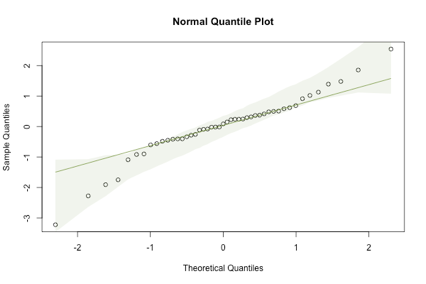 |
| (g) difference in lnRR (C4 vs C3 graminoid) |  | |
| 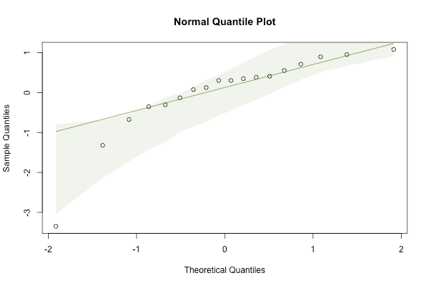 |  |  |

#### **Figure S4**. Normal QQ plots of modelled response variables. (a) The slight negative skewness of lnRR of herbaceous species richness is confirmed, while the rest of the modelled variables (b-g) is generally within the confidence envelopes. Plots were produced using package ecostats v.0.1.4 (Warton, 2020).

#### **
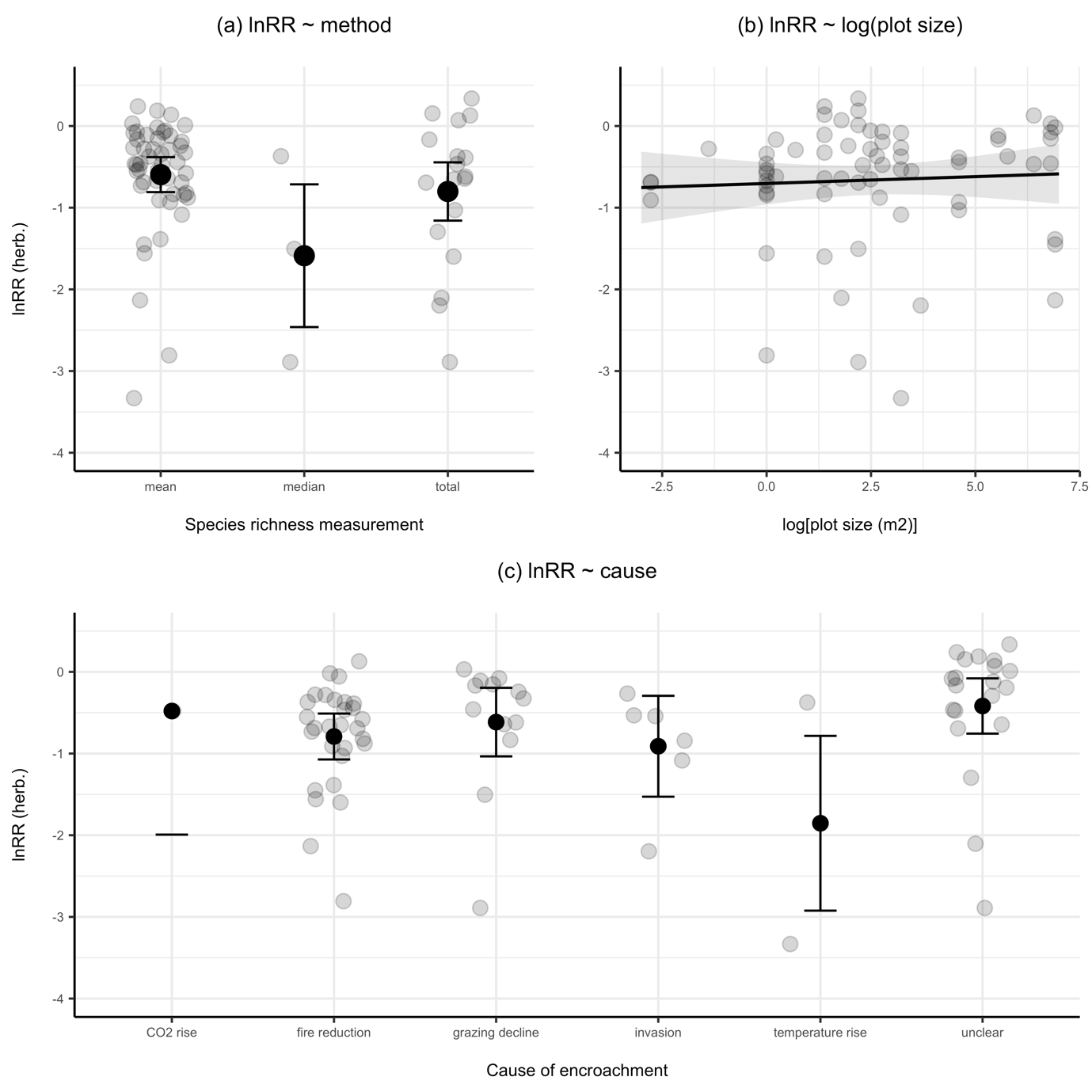
**

#### **Figure S5**. The effect of method of richness calculation, plot size, and cause of encroachment on lnRR (herb.). None of the (a) method of richness calculation (p-value = 0.082), 2) plot size (p-value = 0.650), or 3) cause of encroachment (p-value = 0.161) has a significant impact on the lnRR values. Black dots and segments (a, c) and black solid line with the grey-shaded area (b) are the model predictions with 95%CI. The plot size was logarithmically transformed to achieve normal distribution.

####
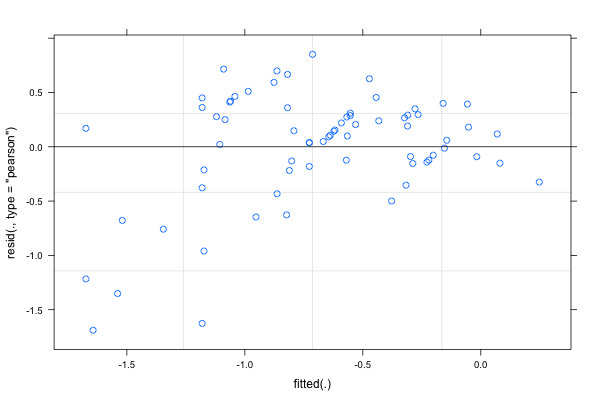


#### **Figure S6**. Homoscedasticity check of the univariate model with the extent of encroachment and random effect of study. The plot shows the residuals vs fitted values. There is a marginal fan-shape pattern in the data, which was expected considering the slight left skewness of lnRR (herb.) data distribution (Figures S3a and S4a). Therefore, a sensitivity analysis removing values lnRR < -2 was undertaken (Figure S9).

#### **
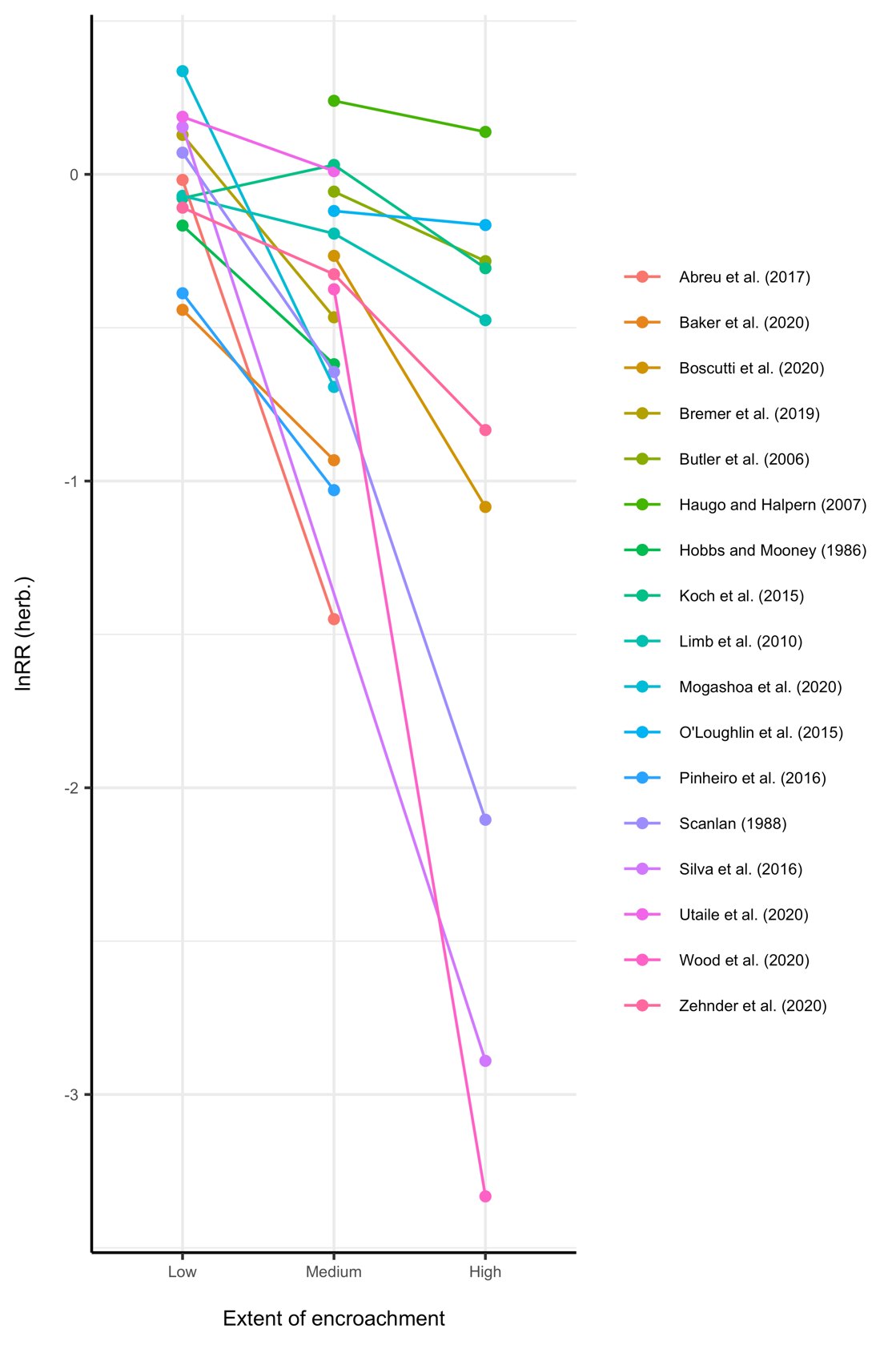
**

#### **Figure S7**. Visualisation of lnRR (herb.) for studies with more than one extent of encroachment. In 17 studies, more than one extent of encroachment was reported. In 16 of them, the lnRR was lower at each higher extent of encroachment.


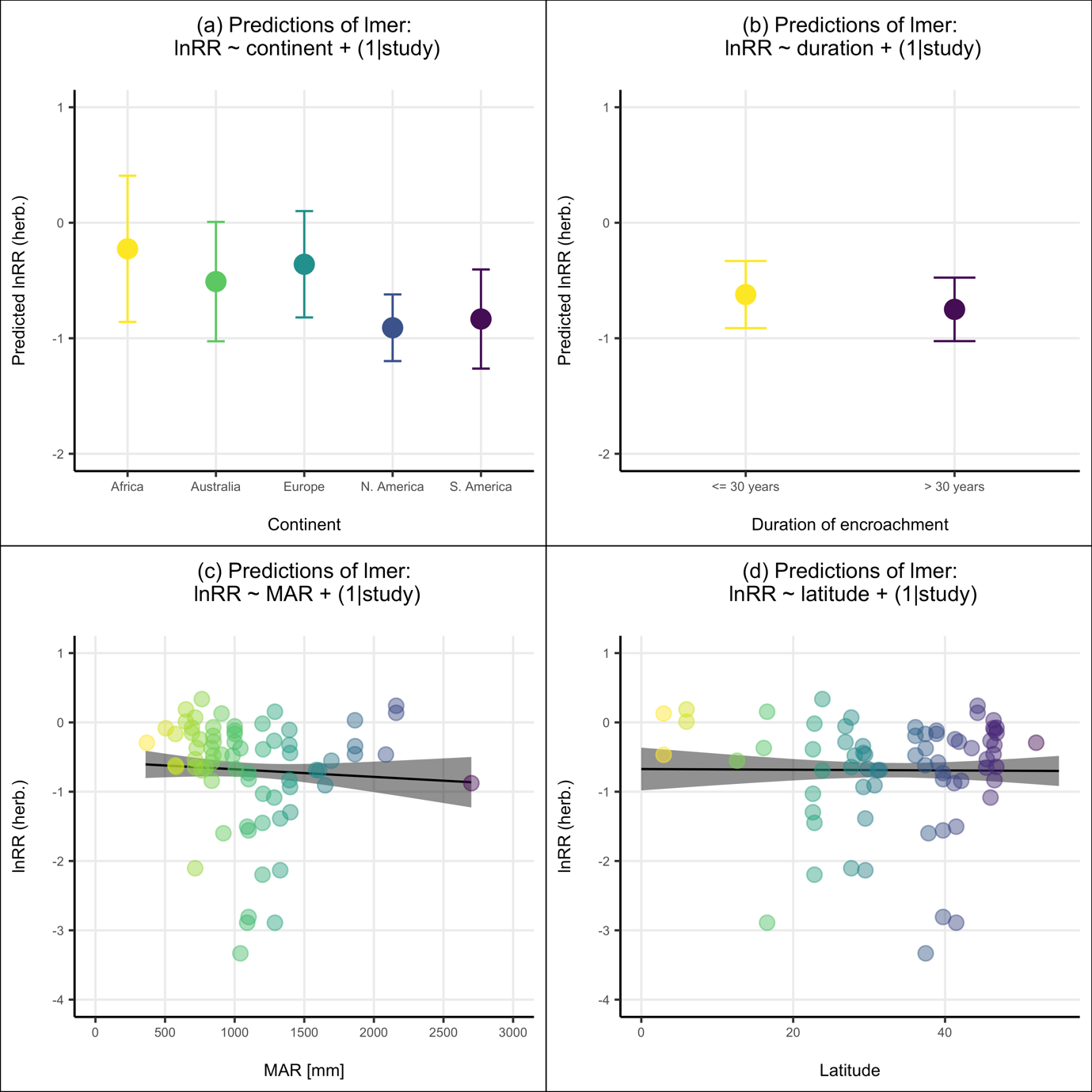


#### **Figure S8.** Predictions of lnRR (herb.) from univariate models with the four remaining fixed effects. In (a) and (b), dots represent model predictions for each category, and solid lines represent 95%CIs. In (c) and (d), coloured points represent raw data points of lnRR (herb.), black lines represent model predictions, and grey shaded areas represent 95%CIs. None of the fixed effects was significant – predictions for each category in (a) and (b) overlapped, while predictions in (c) and (d) had overlapping 95%CIs at both ends of MAR and latitude range respectively. Furthermore, a comparison of each model with a null model with only the random effect of study [lnRR ~ 1 + (1|study)] was conducted with a likelihood ratio test using function anova() which confirmed the lack of significance of fixed effects as p-values were above the 0.05 significance threshold: (a) 0.125, (b) 0.543, (c) 0.617, (d) 0.950.

#### **
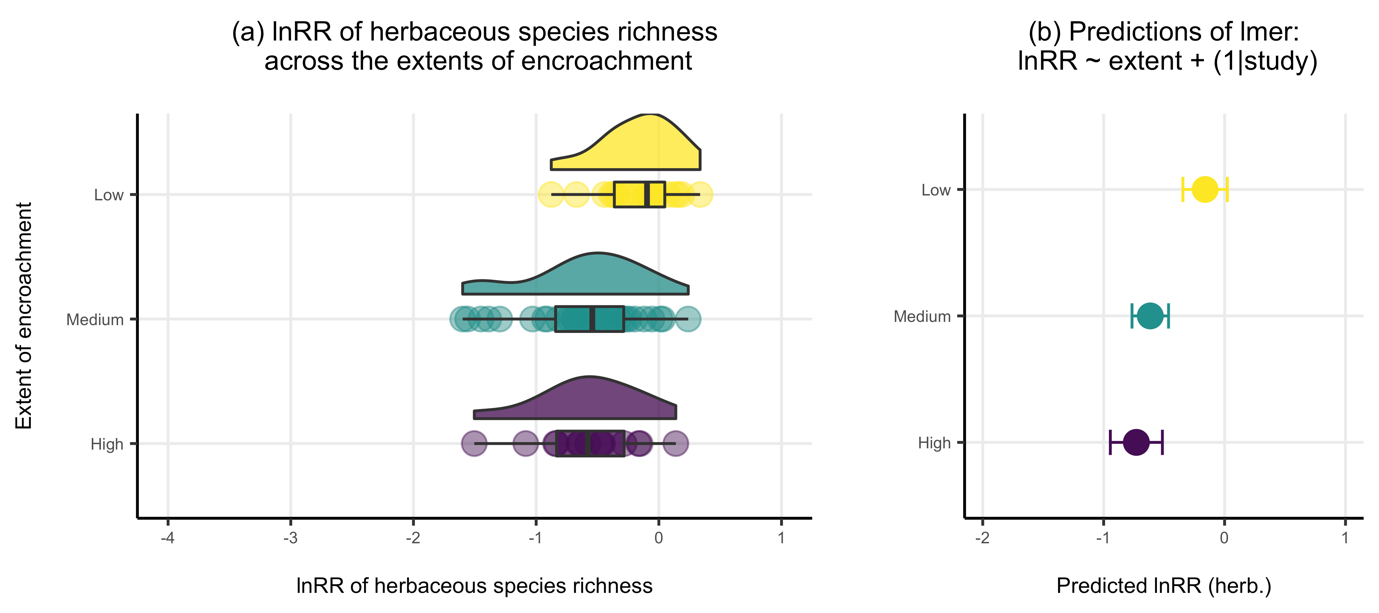
**

#### **Figure S9**. Sensitivity test: examining correlates of herbaceous richness response to encroachment for lnRR > -2. As lnRR was slightly negatively skewed in the initial analysis, we rechecked the linear mixed-effects modelling by removing the lowest lnRR values in order to achieve normal distribution (i.e. only values of lnRR > -2 were used - see (a)). Re-run histogram and normal QQ plot confirmed normal distribution, while the fan-shaped pattern in residual vs fitted values plot was reduced. AIC analysis confirmed that the model with only one fixed effect – the extent of encroachment (lnRR ~ extent + (1|study)) still had the lowest AIC (79.45) and it explained 65.0% of variance; a model with five fixed effects had AIC of ​​121.48 and explained 67.1% of variance. (b) Only for the low extent of encroachment the 95%CIs spanned 0 (lnRR = -0.16, 95%CI: -0.34, 0.02), while the values were significantly negative for medium (lnRR = -0.61, 95%CI: -0.77, -0.46) and high (lnRR = -0.73, 95%CI: -0.94, -0.51) extents. Predictions for medium and high extents of encroachment overlapped; however, they were both significantly lower than the prediction for low extent of encroachment.


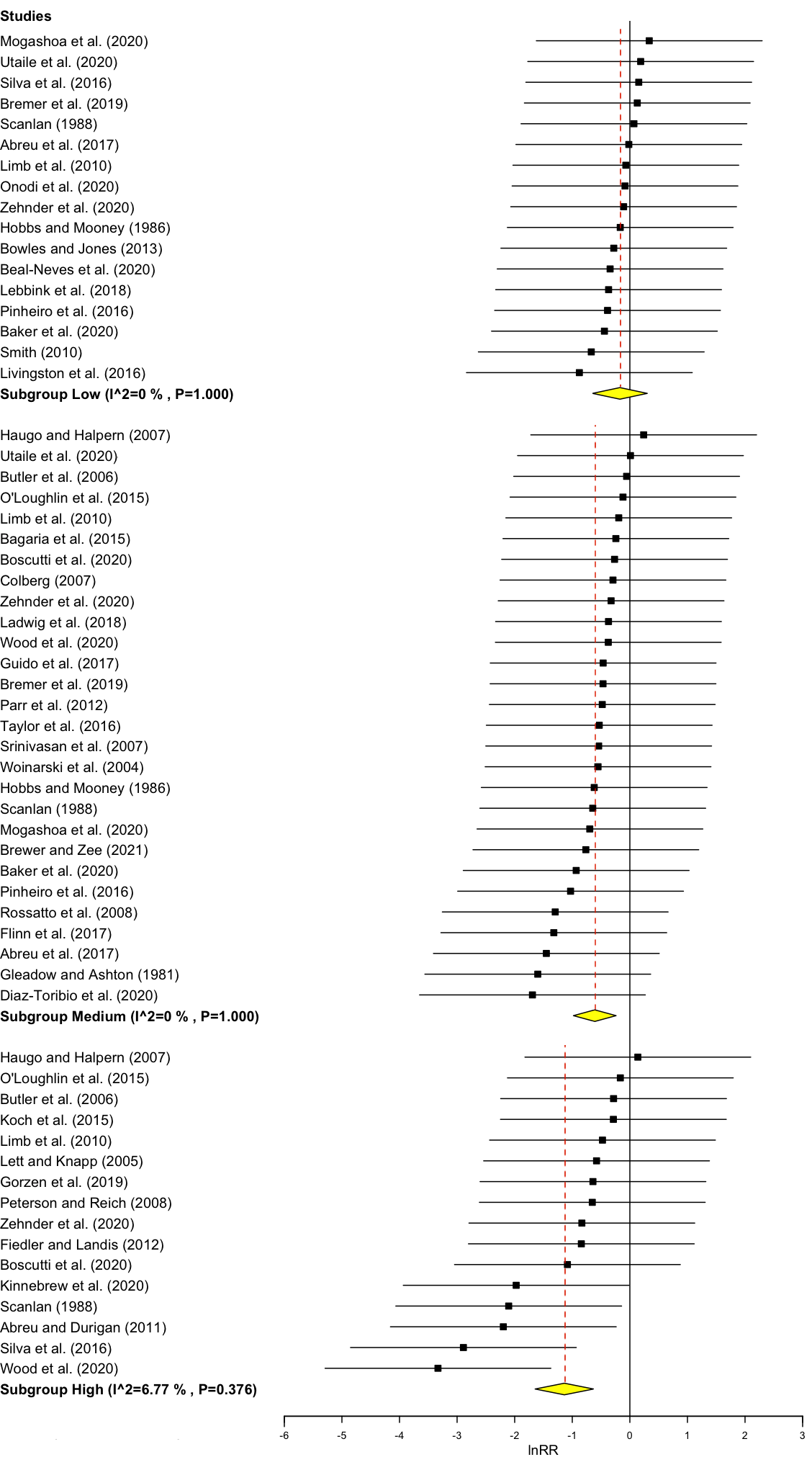


#### **Figure S10**. Sensitivity test: global (un)weighted mean of herbaceous species richness response to the three extents of encroachment. On the left are listed 61 unweighted points of three extent categories from 42 studies (full references in List S2). The studies are ordered by the effect size (from highest to lowest) for each extent category. The size of black squares (effect sizes – lnRR) and the black solid lines are of the same size and length due to equal weighting applied to all studies. Yellow diamonds and red vertical dashed lines represent the weighted mean for each extent category. The mean lnRR was -0.173 (95%CI: -0.648, 0.302) at low extent of encroachment, -0.610 (95%CI: -0.980, -0.239) at medium, and -1.138 (95%CI: -1.645, -0.630). The confidence interval spans 0 for the low extent of encroachment which indicates that the lnRR is not significantly negative for this category of extent.

## References

﻿Fox, J., & Monette, G. (1992). Generalized collinearity diagnostics. *Journal of the American Statistical Association*, 87, 178–183. doi.org/10.1080/01621459.1992.10475190

James, G., Witten, D., Hastie, T., & Tibshirani, R. (2014). *An Introduction to Statistical Learning: With Applications in R.* Springer Publishing Company, Incorporated.

Ratajczak, Z., Nippert, J. B., & Collins, S. L. (2012). Woody encroachment decreases diversity across North American grasslands and savannas. *Ecology*, 93(4), 697–703. doi.org/10.1890/11-1199.1

Viechtbauer, W. (2010). Conducting meta-analyses in R with the metafor package. *Journal of Statistical Software*, 36(3), 1–48. doi.org/10.18637/jss.v036.i03

Warton, D. (2020). *ecostats: Code and Data Accompanying the Eco-Stats Text*. R package version 0.1.4. Retrieved from https://CRAN.R-project.org/package=ecostats
